# Ethanol tolerance of *Clostridium thermocellum*: the role of chaotropicity, temperature and pathway thermodynamics on growth and fermentative capacity

**DOI:** 10.1186/s12934-022-01999-8

**Published:** 2022-12-25

**Authors:** Teun Kuil, Johannes Yayo, Johanna Pechan, Jan Küchler, Antonius J. A. van Maris

**Affiliations:** 1 Department of Industrial Biotechnology, School of Engineering Sciences in Chemistry, Biotechnology and Health, KTH Royal Institute of Technology, Stockholm, Sweden; 2 Present Address: Max Plank Institute for Dynamics of Complex Technical Systems, Otto-Von-Guericke-University Magdeburg, Magdeburg, Germany

**Keywords:** *Clostridium thermocellum*, *Acetivibrio thermocellus*, Chaotropicity, Ethanol tolerance, Temperature, Growth-arrest, *adhE*

## Abstract

**Background:**

*Clostridium thermocellum* is a promising candidate for consolidated bioprocessing of lignocellulosic biomass to ethanol. The low ethanol tolerance of this microorganism is one of the remaining obstacles to industrial implementation. Ethanol inhibition can be caused by end-product inhibition and/or chaotropic-induced stress resulting in increased membrane fluidization and disruption of macromolecules. The highly reversible glycolysis of *C. thermocellum* might be especially sensitive to end-product inhibition. The chaotropic effect of ethanol is known to increase with temperature. This study explores the relative contributions of these two aspects to investigate and possibly mitigate ethanol-induced stress in growing and non-growing *C. thermocellum* cultures.

**Results:**

To separate chaotropic from thermodynamic effects of ethanol toxicity, a non-ethanol producing strain AVM062 (P_*clo1313_2638*_::ldh* ∆*adhE*) was constructed by deleting the bifunctional acetaldehyde/alcohol dehydrogenase gene, *adhE*, in a lactate-overproducing strain. Exogenously added ethanol lowered the growth rate of both wild-type and the non-ethanol producing mutant. The mutant strain grew quicker than the wild-type at 50 and 55 °C for ethanol concentrations ≥ 10 g L^−1^ and was able to reach higher maximum OD_600_ at all ethanol concentrations and temperatures. For the wild-type, the maximum OD_600_ and relative growth rates were higher at 45 and 50 °C, compared to 55 °C, for ethanol concentrations ≥ 15 g L^−1^. For the mutant strain, no positive effect on growth was observed at lower temperatures. Growth-arrested cells of the wild-type demonstrated improved fermentative capacity over time in the presence of ethanol concentrations up to 40 g L^−1^ at 45 and 50 °C compared to 55 °C.

**Conclusion:**

Positive effects of temperature on ethanol tolerance were limited to wild-type *C. thermocellum* and are likely related to mechanisms involved in the ethanol-formation pathway and redox cofactor balancing. Lowering the cultivation temperature provides an attractive strategy to improve growth and fermentative capacity at high ethanol titres in high-cellulose loading batch cultivations. Finally, non-ethanol producing strains are useful platform strains to study the effects of chaotropicity and thermodynamics related to ethanol toxicity and allow for deeper understanding of growth and/or fermentation cessation under industrially relevant conditions.

**Supplementary Information:**

The online version contains supplementary material available at 10.1186/s12934-022-01999-8.

## Background

Achieving net-zero CO_2_ emissions in the transport sector, especially in heavy-duty, difficult-to-electrify transport modes such as shipping, aviation, and long-haul trucking, is expected to require large-scale implementation of biofuels [1–3]. Bioethanol, the biofuel that has the largest annual production volume (ca. 100 billion liters in 2021 [4]), is currently mainly derived from starch- and sugar-based feedstocks, such as corn and sugar cane. However, to meet future bioethanol demands, ethanol derived from renewable cellulosic feedstocks will likely be necessary [5]. Traditional yeast-based lignocellulosic ethanol production requires heavy thermochemical pretreatment and addition of fungal cellulases to liberate the fermentable sugars in the feedstock, which significantly increase production costs [6, 7]. Dramatic cost reduction could potentially be achieved by combining consolidated bioprocessing (CBP), where biomass solubilization and fermentation take place in one process unit without added enzymes, with milling during fermentation (known as cotreatment); a process called C-CBP [7–10].

The anaerobic thermophile *Clostridium thermocellum* (recently renamed as *Acetivibrio thermocellus* [11]) is considered a promising candidate for C-CBP due to its superior capacity to solubilize lignocellulosic biomass [6, 10, 12]. The released sugars are predominantly converted to ethanol, weak organic acids, and hydrogen. To date, *C. thermocellum* has been engineered to produce ethanol at 75% of the theoretical maximum yield [13] with a maximum titre of 30 g L^−1^ [14]. However, for cost-effective lignocellulosic ethanol production, 90% of the theoretical yield and 40 g L^−1^ ethanol are needed [5, 15].

Ethanol tolerance is one of the main titre limitations. In contrast to the efficient ethanol-producers *Zymomonas mobilis* and *Saccharomyces cerevisiae*, which can tolerate up to 127 g L^−1^ [16] and 197 g L^−1^ [17] ethanol, respectively, wild-type *C. thermocellum* is already strongly inhibited at ethanol concentrations of 5 g L^−1^ and can withstand up to 20 g L^−1^ ethanol [18–21]. Furthermore, in high cellulose-loading (> 100 g L^−1^) batch fermentations with wild-type and engineered strains, growth typically ceases at ethanol concentrations of 10–15 g L^−1^, after which fermentation still continues [14, 22, 23]. Ethanol tolerant strains have been created by sequential transfers in medium with increasing ethanol concentrations, resulting in ethanol tolerance up to 50 g L^−1^ [20, 24–26]. However, despite the increased tolerance, no improvements in ethanol productivity were observed for these mutants and a gap between the currently produced titre and the target for industrial implementation remains.

The toxicity of ethanol is strongly related to its chaotropic nature. Chaotropes are compounds that disrupt biological systems by increasing the entropy of individual macromolecules [27]. This leads to denaturation of these macromolecules (proteins, DNA, RNA, lipids) and increased membrane fluidization affecting nutrient transport, ATP generation, and redox cofactor ratios, resulting in cellular stress and potentially cell death [27–29]. A common mechanism observed in *C. thermocellum*, and many other species, to overcome chaotropic-induced ethanol stress is to change the membrane fatty acid composition, leading to increased membrane rigidity [29–31]. Furthermore, changes in membrane protein profiles have also been observed in *C. thermocellum* [20].

In addition to chaotropic effects, end-product inhibition of glycolytic and ethanologenic enzymes caused by intracellular accumulation of redox cofactors and metabolites is commonly observed [32–35]. Interestingly, genome resequencing and characterization of ethanol-adapted *C. thermocellum* strains have identified mutations in the bifunctional acetaldehyde/alcohol dehydrogenase gene, *adhE*, that alter the cofactor specificity from NADH to NADPH [24, 36]. Subsequent introduction of the mutant *adhE* in wild-type *C. thermocellum* conferred the mutant phenotype and enabled growth up to 40 g L^−1^ ethanol [25]. Hence, thermodynamic limitations of the ethanol formation pathway at increased ethanol concentration appear to be a dominant mechanism for inhibition in *C. thermocellum*.

Apart from constructing more ethanol-tolerant mutant strains, optimization of process conditions, specifically cultivation temperature, could play an important role in improving ethanol tolerance. Previous studies with various yeast and bacterial species have shown that decreasing the cultivation temperature is an effective way to improve ethanol tolerance and productivity [18, 28, 37–40]. Similar to the membrane changes observed for ethanol-adapted mutants, lower temperature alters the membrane composition thereby decreasing membrane fluidity and permeability [41]. Hence, temperature reduction could be an efficient strategy to combat chaotropic-induced stresses and improve ethanol tolerance and productivity of *C. thermocellum*.

The aim of the present study is to investigate if lowering the cultivation temperature can mitigate the chaotropic-induced ethanol stress in *C. thermocellum*. To understand if chaotropicity plays an important role in *C. thermocellum*, a non-ethanol producing mutant strain was first constructed to separate the thermodynamic from biophysicochemical effects of ethanol. Subsequently, the wild-type and mutant strain were characterized in batch bottle cultivations grown in the presence of up to 50 g L^−1^ exogenously added ethanol at cultivation temperatures ranging from 45 to 55 °C. Finally, the influence of cultivation temperature on the fermentative capacity of growth-arrested cells was tested at various added ethanol concentrations.

## Results

### Construction of a non-ethanol producing mutant strain

Previous work has demonstrated that to construct a non-ethanol producing strain, deletion of the bifunctional acetaldehyde/alcohol dehydrogenase gene, *adhE*, is necessary [42]. Attempts to delete this gene in wild-type strain DSM1313 were unsuccessful. Given that ethanol is one of the major fermentation products in DSM1313 (Table 1), removing this pathway would require the metabolism to shift to other catabolic end-products (e.g., lactate, formate, hydrogen, and acetate). However, it is likely that these alternative product pathways have limited capacity to balance all redox cofactors in a wild-type strain. In line with this notion, a previously constructed *adhE* knockout strain acquired a point mutation in the *ldh* gene which removed the allosteric activation of lactate dehydrogenase by fructose 1,6-bisphosphate thereby likely increasing its capacity to carry flux and balance redox cofactors [42]. Therefore, in this study a lactate dehydrogenase overexpressing strain, called AVM002 (P_*clo1313_2638*_::ldh*), was first constructed by integrating the native *ldh* gene behind the strong constitutive *clo1313_2638* promoter [43] in DSM1313. Using this strain as mother strain, *adhE* could be deleted, yielding strain AVM062 (P_*clo1313_2638*_::ldh* ∆*adhE*).

**Table 1:** Maximum specific growth rate (µ^max^), biomass and fermentation product yields (Y), and lactate dehydrogenase (Ldh) activity of DSM1313 and AVM062

Strain	DSM1313	AVM062
Relevant genotype	Wild-type	P_*clo1313_2638*_::ldh* ∆*adhE*
µ^max^ (h^−1^)	0.35 ± 0.00	0.24 ± 0.00
Y_biomass/cellobiose_ (g_x_ g^−1^)	0.27 ± 0.00	0.21 ± 0.00
Y_ethanol/cellobiose_ (mol mol^−1^)	0.76 ± 0.07	< 0.01
Y_acetate/cellobiose_ (mol mol^−1^)	0.98 ± 0.00	0.82 ± 0.02
Y_formate/cellobiose_ (mol mol^−1^)	0.25 ± 0.00	0.02 ± 0.00
Y_lactate/cellobiose_ (mol mol^−1^)	0.18 ± 0.01	1.63 ± 0.03
Ldh activity (µmol mg_protein_^−1^ min^−1^)	0.57 ± 0.05	30.46 ± 1.90

Batch serum bottle cultures were grown on modified LC medium containing 10 g L^−1^ cellobiose. Averages and mean deviations were obtained from independent biological duplicates. The detection limit for the ethanol yield was 0.01 mol mol^−1^

To investigate the impact of overexpressing *ldh* and knocking out *adhE*, the maximum specific growth rate, biomass yield, fermentation product yields and lactate dehydrogenase activity of AVM062 were determined in batch serum bottle cultures (Table 1). Removal of *adhE* completely abolished ethanol formation in AVM062 (P_*clo1313_2638*_::ldh* ∆*adhE*). Furthermore, overexpression of *ldh* increased the lactate dehydrogenase activity with ca. 50-fold and resulted in a ninefold increase in the lactate yield (0.18 mol mol^−1^ and 1.63 mol mol^−1^, respectively) compared to DSM1313. Finally, the growth rate and biomass yield were lowered to 0.24 h^−1^ and 0.21 g_x_ g^−1^, respectively, which was ca. 31% (*P* < 0.01) and 22% (*P* < 0.01) lower than that of DSM1313. Overexpression of *ldh* allowed the lactate pathway to carry sufficient flux to at least compensate for most of the loss of ethanol formation and provides a platform strain in which glycolysis is not affected by end-product inhibition of ethanol.

### Effect of cultivation temperature on the ethanol tolerance of growing cells

To investigate if chaotropicity of ethanol plays a role in *C. thermocellum* and to study if this can be counteracted by decreasing the cultivation temperature, both the wild-type (DSM1313) and the non-ethanol producing mutant (AVM062) were grown in the presence of various added ethanol concentrations at 55, 50, and 45 °C.

**Fig. 1:**
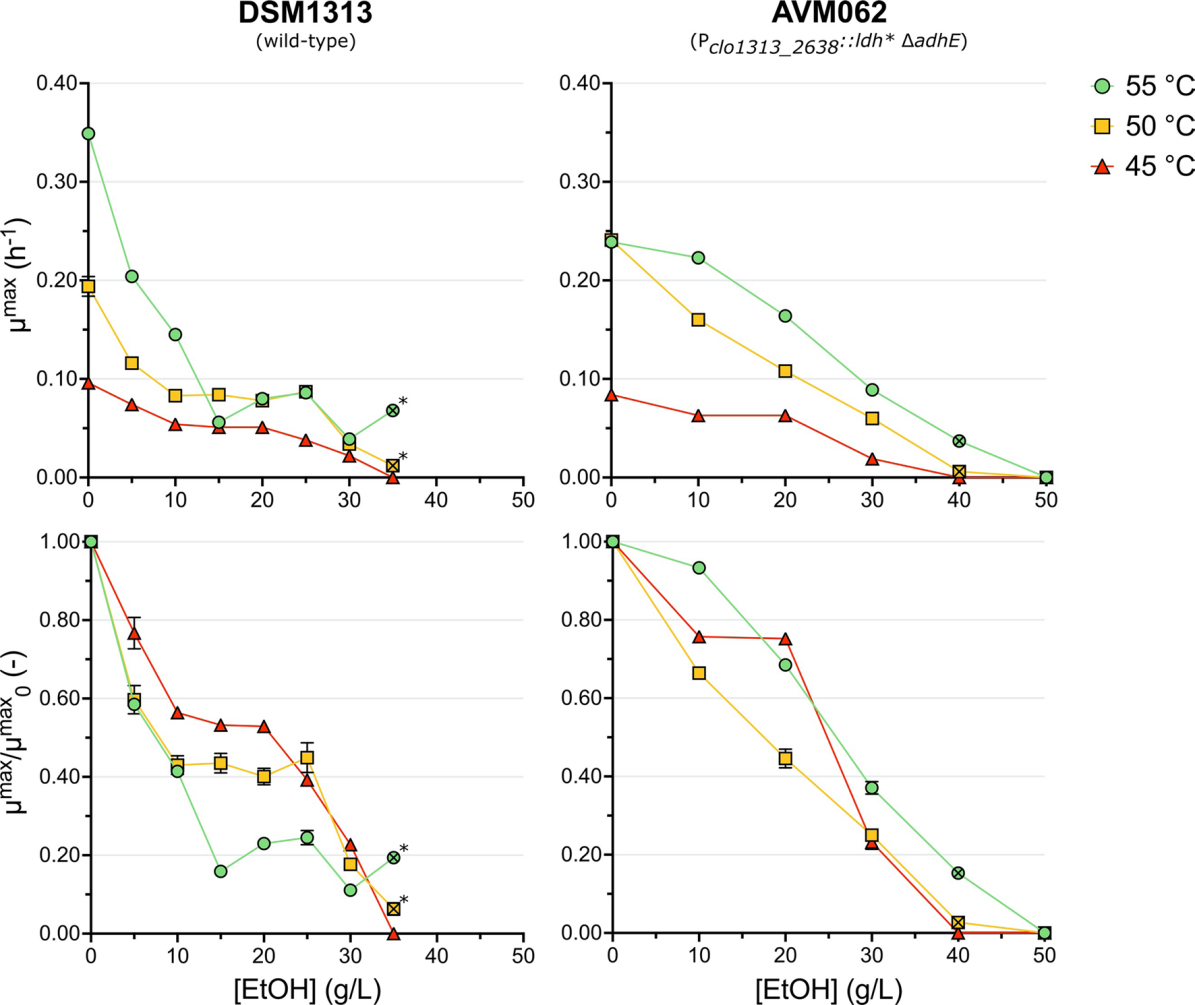
Maximum specific growth rate and relative growth rate of DSM1313 (wild-type; left column) and AVM062 (P_*clo1313_2638*_::ldh* ∆adhE; right column) as a function of the added ethanol concentration at 55, 50 and 45 °C. The relative growth rate (µ^max^/µ^max^_0_) is defined as the maximum specific growth rate in the presence of added ethanol (µ^max^) divided by the maximum specific growth rate in the absence of added ethanol (µ^max^_0_) at the same temperature. Batch serum bottle cultures were grown on modified LC medium with 10 g L^−1^ cellobiose. The crossed-out symbols are used for cultures that started growing exponentially after > 40 h. For DSM1313 grown at 50 and 55 °C in the presence of 35 g L^−1^ added ethanol only three points were included for the µ calculation (indicated with an *). Averages and mean deviations were obtained from independent biological duplicates. Absence of error bars indicates mean deviations were smaller than the symbol size

**Fig. 2:**
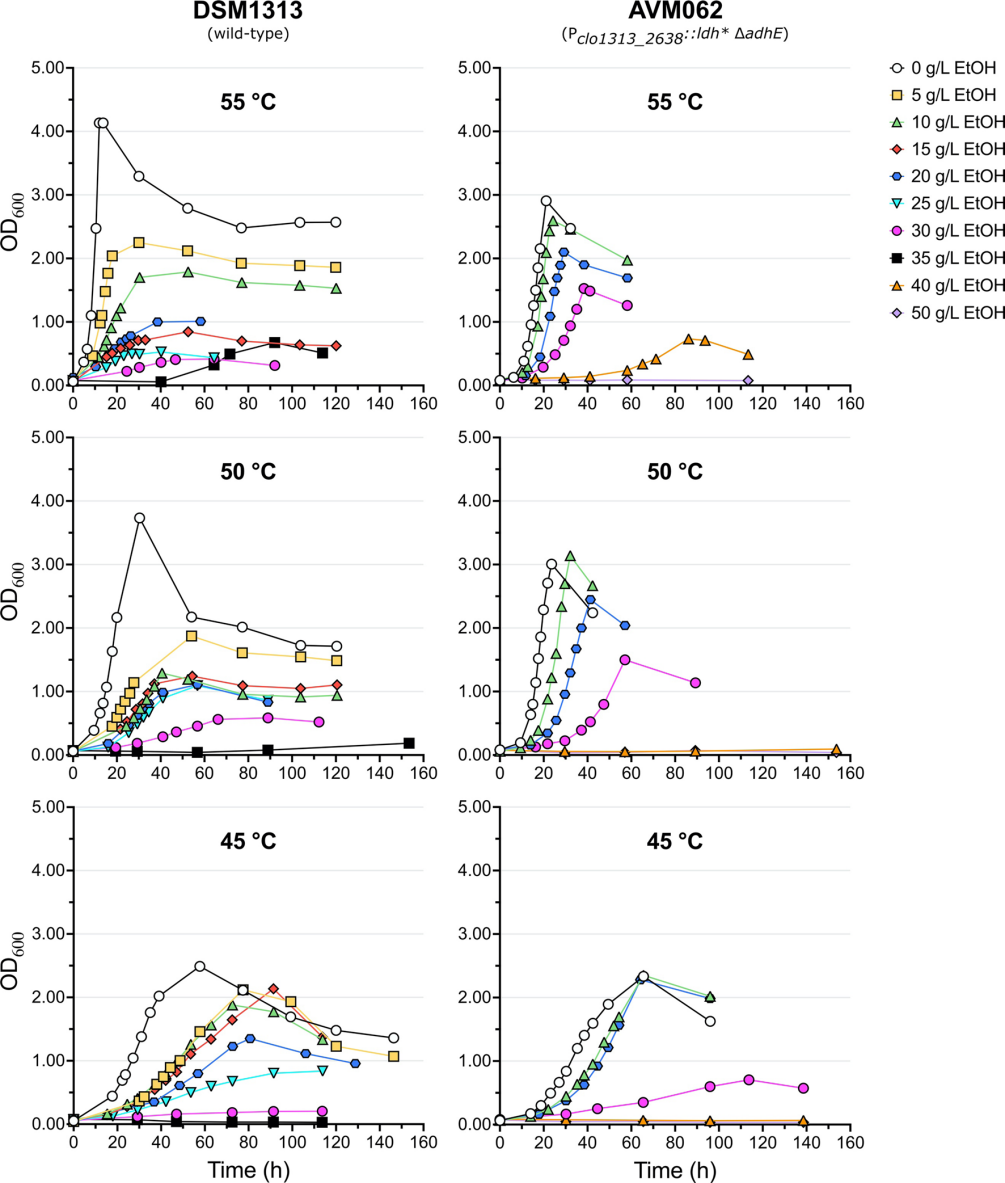
Growth of DSM1313 (wild-type; left column) and AVM062 (P_*clo1313_2638*_::ldh* ∆adhE; right column) as a function of the added ethanol concentration at 55, 50 and 45 °C. Batch serum bottle cultures were grown on modified LC medium with 10 g L^−1^ cellobiose. Data are shown for one representative experiment (*n* = 2)

At all temperatures DSM1313 was able to grow without a lag phase up to an added ethanol concentration of 30 g L^−1^. When grown with 35 g L^−1^ added ethanol growth was only observed > 40 h after inoculation at 55 and 50 °C, whilst no growth was observed at 45 °C (Figs. 1 and 2). For AVM062 (P_*clo1313_2638*_::ldh* ∆*adhE*), growth was observed up to 40 g L^−1^ added ethanol for 55 and 50 °C, albeit only beyond 40 h after inoculation, whereas at 45 °C growth was detected up to 30 g L^−1^ added ethanol.

The addition of ethanol lowered the growth rate and maximum OD_600_ for both strains at all cultivation temperatures compared to the control without added ethanol. For the wild-type strain DSM1313 growth was equal at 55 and 50 °C for added ethanol concentrations ≥ 15 g L^−1^ (Fig. 1). Furthermore, for the same strain, the relative growth rate (defined as the maximum specific growth rate in the presence of added ethanol (µ^max^) divided by the maximum specific growth rate in the absence of added ethanol (µ^max^_0_)) increased at 45, and 50 °C compared to 55 °C. Additionally, the maximum OD_600_ for the wild-type at ethanol concentrations above 10 g L^−1^ increased with lower cultivation temperatures (Fig. 2). These observations indicate that ethanol-induced damages can be counteracted by lowering the cultivation temperature and hint at a chaotropic effect of ethanol in *C. thermocellum*. Interestingly, the positive effect of lowering the cultivation temperature on the absolute and relative growth rate or maximum OD_600_ were not observed in AVM062 (Figs. 1 and 2).

The non-ethanol producing strain AVM062 (P_*clo1313_2638*_::ldh* ∆*adhE*) is much more ethanol tolerant than wild-type DSM1313 (Fig. 1). At 55 °C, the growth rate of DSM1313 already dropped by ca. 40% at only 5 g L^−1^ added ethanol compared to the control, while a similar drop was observed for the non-ethanol producing mutant (AVM062) at ethanol concentrations above 20 g L^−1^. Furthermore, AVM062 can grow quicker than the wild-type (DSM1313) at 55 and 50 °C for added ethanol concentrations ≥ 10 g L^−1^. This mutant strain also reached a much higher maximum OD_600_ at all added ethanol concentrations regardless of the cultivation temperature (Fig. 2). These results indicate that chaotropicity of ethanol plays a role in *C. thermocellum*, however end-product inhibition of ethanol seems dominant and largely determines the ethanol tolerance of wild-type *C. thermocellum*; an observation that is consistent with earlier reports obtained with different methods [19, 25, 32].

**Fig. 3:**
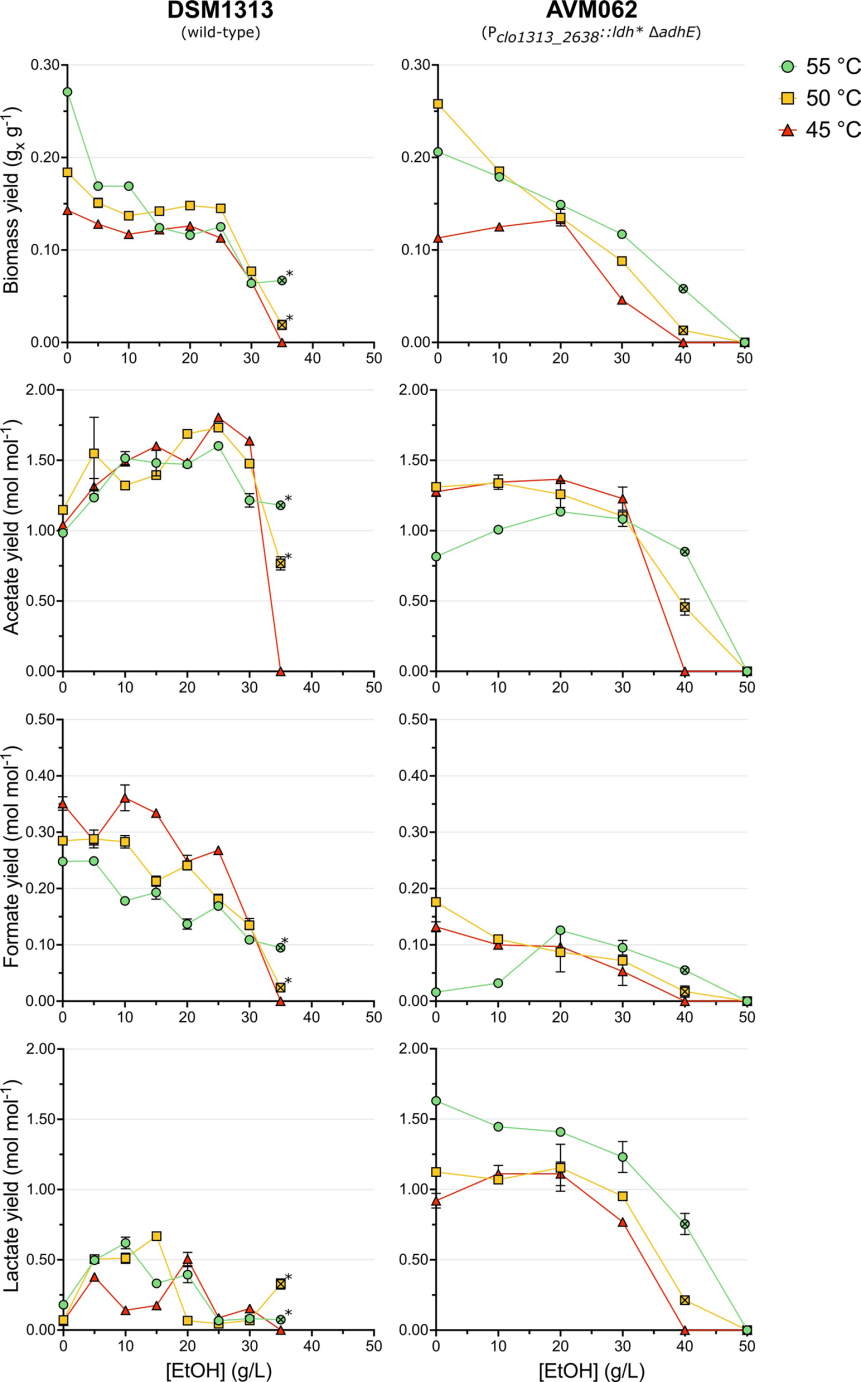
Biomass yield and fermentation product yields of DSM1313 (wild-type; left column) and AVM062 (P_*clo1313_2638*_::ldh* ∆adhE; right column) as a function of the added ethanol concentration at 55, 50 and 45 °C. Batch serum bottle cultures were grown on modified LC medium with 10 g L^−1^ cellobiose. Produced ethanol yields could not be accurately determined for cultures with ≥ 10 g L^−1^ added ethanol and are not reported. The crossed-out symbols are used for cultures that started growing exponentially after > 40 h. For DSM1313 grown at 50 and 55 °C in the presence of 35 g L^−1^ added ethanol only three points were included for the yield calculation (indicated with an *). Averages and mean deviations were obtained from independent biological duplicates. Absence of error bars indicates mean deviations were smaller than the symbol size

In addition to the effects observed on growth, increasing ethanol concentrations also affected the biomass- and fermentation product yields of DSM1313 and AVM062 (P_*clo1313_2638*_::ldh* ∆*adhE*) (Fig. 3). The biomass yield followed the same trend as the growth rate for both strains, which decreased at increasing ethanol concentrations and showed, for the wild-type DSM1313, a shift in optimum temperature from 55 to 50 °C for ethanol concentrations ≥ 15 g L^−1^. Furthermore, the formate yield decreased with increasing ethanol concentrations, and the acetate yield increased up to 25 g L^−1^ added ethanol for all temperatures in DSM1313. Interestingly, the acetate yield only increased slightly at 55 °C (ca. 1.3-fold) and stayed relatively constant up to 30 g L^−1^ added ethanol at 50 and 45 °C in AVM062 (P_*clo1313_2638*_::ldh* ∆*adhE*). This result indicates that the increase in acetate yield for the wild-type at higher added ethanol concentrations can primarily be attributed to an intracellular build-up of acetyl-CoA and concomitant shift in fermentation end-products resulting from end-product inhibition of ethanol. No consistent pattern was observed for the lactate yield. Unfortunately, the trends in ethanol yield could not be accurately determined for added ethanol concentrations ≥ 10 g L^−1^ as the produced ethanol concentration was too small compared to the added ethanol concentration.

Finally, for all temperatures and for both the wild-type strain DSM1313 and the non-ethanol producing mutant AVM062, in the presence of added ethanol growth generally ceased before all cellobiose was completely consumed (Additional files 2 and 3). The residual amount of cellobiose after growth stopped also increased with increasing added ethanol concentrations. After growth stopped, large parts of cellobiose were hydrolyzed to glucose, while the rest was continuously fermented to mainly acetate and lactate.

### Effect of cultivation temperature on the ethanol tolerance of growth-arrested cells

In yeast-based industrial bioethanol processes, especially towards the end of the cultivation, growth ceases when the ethanol concentration increases above a critical threshold [27]. After this point, and similar to what has been observed in this study, cells continue to ferment the remaining sugars to the desired product. Considering that continued fermentation has also previously been observed for high-cellulose loading batch fermentations with wild-type and engineered *C. thermocellum* [14, 22, 23], we investigated if lowering the cultivation temperature could improve the fermentative capacity of growth-arrested wild-type *C. thermocellum* cells in the presence of various added ethanol concentrations.

To test this, wild-type cells were harvested from exponentially growing cultures, washed, and transferred to a previously designed sulfur-limited LC medium [44] containing 0, 15, 30, and 40 g L^−1^ added ethanol. The upper limit of 40 g L^−1^ added ethanol was chosen as this is commonly reported as the minimum concentration needed for an economically viable bioethanol process from lignocellulosic biomass [5, 15]. The sulfur-limited medium allows for a short period of initial growth, where the remaining sulfur sources (mainly cysteine) are consumed, followed by a period of growth-arrest where cells continuously ferment cellobiose to mainly acetate and lactate, and in the absence of added ethanol, to ethanol as main fermentation products (Additional file 4). Given that in *C. thermocellum* carbon balances of cultivations are known to close poorly (up to 60–90%) [14, 22, 44–46], using the measured fermentation products as a proxy for the fermentative capacity of growth-arrested cells would result in a significant underestimation of this value. Therefore, the fermentative capacity was determined from the change of the fermented cellobiose concentration, which is calculated by assuming that all cellobiose consumed by growth-arrested cells minus the cellobiose that is hydrolyzed to glucose is fermented. The change of the fermented cellobiose concentration and the biomass concentration are subsequently used to calculate a biomass-specific cellobiose fermentation rate, which represents the fermentative capacity of growth-arrested cells (see Methods for details).

**Fig. 4:**
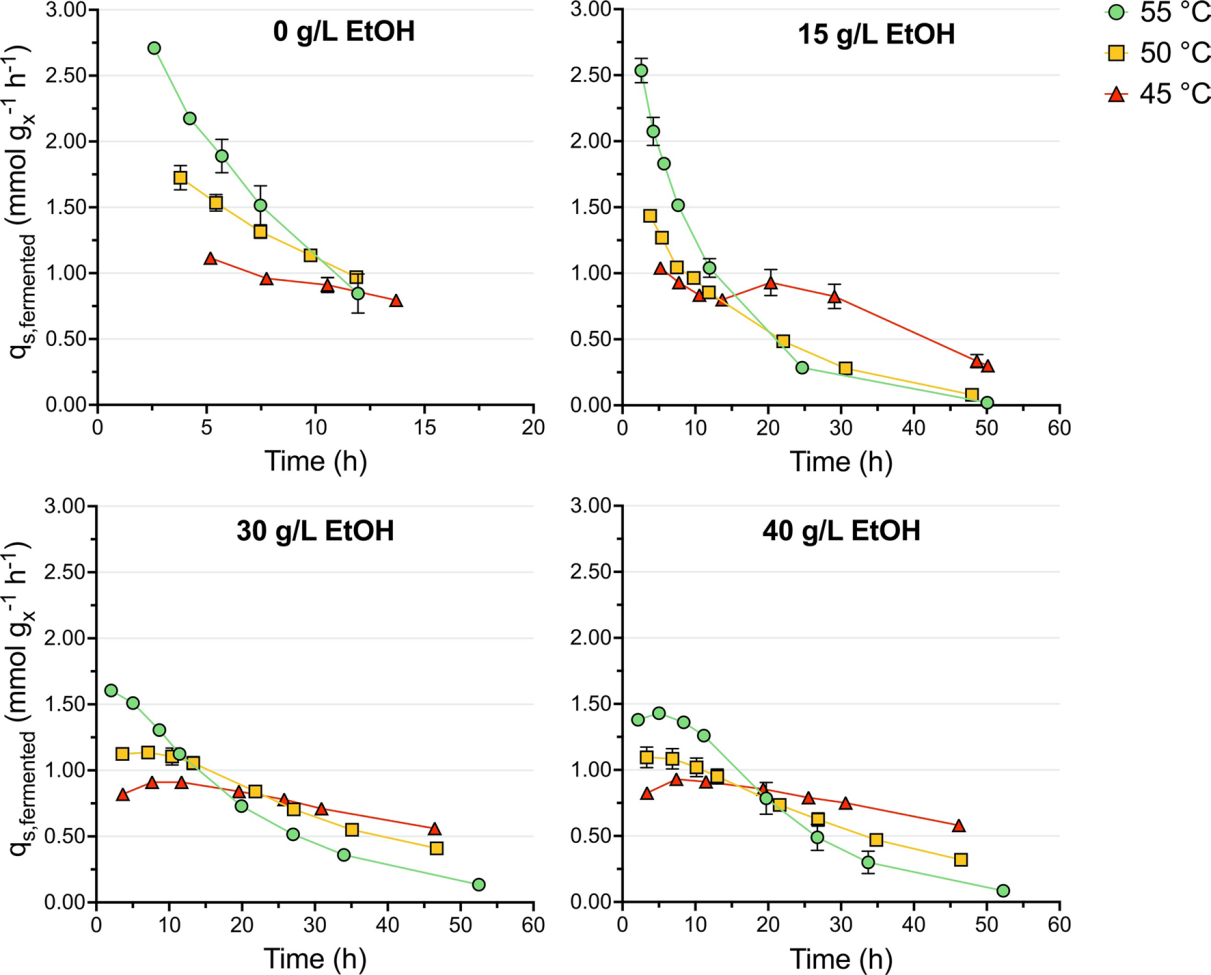
Biomass-specific cellobiose fermentation rate of DSM1313 as a function of time in the presence of 0, 15, 30, and 40 g L^−1^ added ethanol during growth-arrest studies. Batch serum bottle cultures were grown on modified LC medium without Na_2_SO_4_ and with 0.01 g L^−1^ cysteine and 10 g L^−1^ cellobiose. The biomass-specific cellobiose fermentation rate was calculated by correcting for cellobiose that was hydrolyzed to glucose and assuming that the rest of the consumed cellobiose is fermented (see Methods for details). Data is plotted from the moment growth was arrested until cellobiose became limiting (> 0.5 mM) (Additional files 4 and 5). Averages and mean deviations were obtained from independent biological duplicates. Absence of error bars indicates mean deviations were smaller than the symbol size

For the first 12–15 h, growth-arrested cells maintained the highest specific fermentation rate for all added ethanol concentrations at 55 °C compared to 45 and 50 °C (Fig. 4). After this initial period, the specific fermentation rate continued to rapidly decrease at 55 °C, while this decrease was much slower at the lower temperatures. Furthermore, for this second period, the highest rates were achieved at 45 °C for all added ethanol concentrations. Similar trends were observed for the specific formate and acetate production rates (Additional file 6). Specific lactate production rates initially increased for all conditions, reaching a maximum, and decreased afterwards (Additional file 6). Over time, the highest specific lactate production rates were achieved at 45 and/or 50 °C. Specific glucose production rates were highest at 55 °C for all conditions during the entire duration of the experiment and increased at higher ethanol concentrations. As expected, the specific total cellobiose consumption rates showed similar trends as the specific fermentation rate (Additional file 6 and Fig. 4). These results indicate that lowering the cultivation temperature is beneficial for the fermentative capacity of growth-arrested cells in the presence of added ethanol.

## Discussion

This study investigated the effect of cultivation temperature on the ethanol tolerance of growing and non-growing cells of *C. thermocellum*. For wild-type DSM1313, lower cultivation temperatures improved the ethanol tolerance as reflected in an increased relative growth rate and maximum OD_600_. Furthermore, the optimum growth temperature (for the tested temperatures) decreased in the presence of exogenous ethanol. This is in line with observations in the related wild-type strain ATCC27405 [18, 21]. Interestingly, growth without a lag phase was detected for the wild-type up to 30 g L^−1^ at all tested temperatures, which is considerably higher than the commonly reported tolerance limit of 20 g L^−1^ [18–21]. After 40 h, growth was even detected up to 35 g L^−1^ at 50 and 55 °C. Ethanol tolerance studies of Biswas et al. [47] have reported minor growth of DSM1313 up to 40 g L^−1^ and Tian et al. [32] previously reported that gradual addition of ethanol allowed growth up to 45 g L^−1^ for DSM1313. Although variations in the literature could occur due to the differences in wild-type strain, inoculation procedures, initial OD_600_, medium composition, and ethanol tolerance setup (i.e., pulse addition, gradual addition, or direct inoculation into medium with high ethanol concentrations), another possible reason for the discrepancies is that the actual tolerance limit of wild-type strains are not truly explored [25, 32] or not easily observed within the time frame of the experiment [19]. The present study shows that wild-type *C. thermocellum* can grow for ca. 2–3 generations at very low growth rates (0.04 h^−1^; doubling time of > 17 h) for ethanol titres ≥ 30 g L^−1^, which could easily be overlooked.

The non-ethanol producing mutant AVM062 (P_*clo1313_2638*_::ldh* ∆*adhE*) was considerably more ethanol tolerant than the wild-type at all temperatures. In addition to the bifunctional acetaldehyde/alcohol dehydrogenase, *adhE*, *C. thermocellum* encodes for several alcohol dehydrogenases (*clo1313_2130*, *clo1313_1827*, *clo1313_0166*, *clo1313_1833*, and *clo1313_0076*) and one aldehyde dehydrogenase (*clo1313_2911*), potentially allowing for indirect conversion of acetyl-CoA into ethanol. However, all these genes are low or moderately expressed in transcriptomic and proteomic studies [14, 48, 49] and none show significant changes in expression upon ethanol addition [33]. Furthermore, deletion of *adhE* completely abolished ethanol formation (this study and [42]) and removed NAD(P)H-dependent aldehyde and alcohol dehydrogenase activity [36, 42]. Hence, activity of AdhE is the dominant (and likely only) mechanism for conversion of acetyl-CoA to ethanol. Therefore, given that alternative product pathways would be able to carry sufficient flux for growth, deletion of the *adhE* gene was expected to remove end-product inhibition and increase the ethanol tolerance. The higher ethanol tolerance observed for the mutant strain is in line with previous reports demonstrating that mutations in *adhE*, resulting in alternative cofactor usage, or complete removal of the gene, conferred the ethanol-tolerant phenotype observed in ethanol-adapted mutant strains [19, 25, 47]. In contrast to the wild-type, lower temperatures did not improve ethanol tolerance in the mutant. This seemingly contradicts the hypothesis that by not having end-product inhibition, chaotropic-induced ethanol effects (i.e., increased membrane fluidization and disruption of macromolecules [27–29]) would be dominant, which would have resulted in mitigation of these effects at lower temperatures. Although temperature-dependent kinetic limitations of lactate dehydrogenase, which the non-ethanol producing strain is heavily relying on, cannot be excluded, the absence of a positive effect of lower temperature in AVM062, narrows down the possible mechanisms underlying the improvements observed for wild-type *C. thermocellum* at lower temperature.

The main genetic difference between the wild-type and mutant strain is *adhE* and thereby the presence of a functional ethanol formation pathway. This means that the observed temperature-induced improvements in the wild-type are likely related to the enzymes required for this pathway to function. The conversion of cellobiose to ethanol and other catabolic end-products (e.g., acetate, formate, and hydrogen) in wild-type cells relies not only on the enzymes directly used in product formation (such as AdhE), but also on an intricate redox network involving various (membrane-bound) ferredoxin:NAD(P)^+^ oxidoreductases and (membrane-bound) hydrogenases that balance the cofactors used in these pathways (i.e., NAD(P)H and reduced ferredoxin) [6, 50]. The enzymes involved in this network can be directly inhibited by ethanol via a chaotropic effect or indirectly via the build-up of NADH/NAD^+^ ratios due to mass action effects at high ethanol titres [32, 51]. Given that this mass-action effect is temperature-independent, the temperature-induced improvement for the wild-type is likely related to the mitigation of the chaotropic effect of ethanol on these enzymes. In contrast, the non-ethanol producing mutant strain relies primarily on NADH redox balancing through lactate formation (ca. 40% of the available electrons from cellobiose end up here) and (likely) is not as dependent on this complex redox network as the wild-type. In view of these results, conversion of cellobiose to ethanol via a simplified (redox) metabolism (e.g., via a pyruvate decarboxylase-based pathway) could not only improve ethanol production, but also provide a strain with better ethanol tolerance.

In addition to the observed positive effects of lower temperature on growth of wild-type DSM1313, growth-arrested cells also demonstrated higher fermentative capacity over time at lower temperatures in the presence of added ethanol. Given that cessation of growth and fermentation are typically reported for wild-type and engineered *C. thermocellum* strains grown at high-cellulose loadings [14, 22, 23], lowering the temperature when growth slows down towards the end of the cultivation could be beneficial for improved ethanol tolerance and productivity under those conditions. However, since lowering the temperature negatively impacts absolute growth rates, thereby increasing running time and costs, it is important to design a temperature profile where growth and fermentation in the absence of growth can both run at their respective optimal conditions.

The elimination of end-product inhibition in the non-ethanol producing mutant makes this a valuable platform strain to further study ethanol inhibition at high-cellulose loadings. As mentioned above, *C. thermocellum* often stops growing at concentrations (10–15 g L^−1^) well below its tolerance limit. This phenomenon, referred to as the “titre gap” [52], is largely explained by the metabolic imbalances resulting from end-product inhibition. However, as discussed by Olson et al. [53], ethanol inhibition might not be the only reason for growth cessation under these conditions (e.g. nutrient limitations, salt inhibition, toxicity of (un)known by-products, etc.). Diagnostic use of the non-ethanol producing mutant might make it possible to distinguish which factors influence growth and/or fermentation cessation under industrially relevant conditions.

## Conclusions

This work demonstrated that lower cultivation temperatures improve the relative growth rate, maximum OD_600_, and fermentative capacity of ethanol-challenged wild-type *C. thermocellum*. Deletion of *adhE* enables a much higher ethanol tolerance than the wild-type, however no positive impact of lowering the cultivation temperature is observed in this strain background. Temperature-induced mitigation of chaotropic ethanol stress is likely related to the ethanol formation pathway and redox cofactor balancing in the wild-type strain. The use of lower cultivation temperatures provides an attractive strategy to improve ethanol production for growth-arrested cells during high-cellulose loading batch cultivations. Non-ethanol producing strains are valuable platform strains to study thermodynamic and chaotropic effects of ethanol and allow for deeper understanding of growth and/or fermentation cessation under industrially relevant conditions.

## Methods

### Strains and maintenance

All *C. thermocellum* strains used in this study (Table 2) originate from DSM1313 (Deutsche Sammlung von Mikroorganismen und Zellkulturen GmbH, Braunschweig, Germany; GenBank accession number: CP002416). Stock cultures were grown anaerobically in CTFUD medium [54]. *Escherichia coli* BL21 derivative cultures (New England Biolabs catalog number C2566I; purchased from BioNordika AB, Solna, Sweden) for plasmid cloning were propagated in LB medium (10 g L^−1^ peptone, 5 g L^−1^ yeast extract, 10 g L^−1^ NaCl) supplemented with 5 µg mL^−1^ chloramphenicol. All strains were stored in 1-mL aliquots in cryogenic vials (VWR International AB, Stockholm, Sweden) at − 80 °C, after addition of sterile glycerol (25%, vol/vol) to overnight cultures. For *C. thermocellum*, stocking was done in a vinyl anaerobic chamber from Coy Laboratory Products, containing 5% H_2_, 10% CO_2_, and 85% N_2_ (Strandmöllen AB, Ljungby, Sweden).

**Table 2:** Strains used in this study

Strain name	Parental strain	Organism	Relevant genotype	Source
*E. coli* T7 Express		*E. coli*	*fhuA2 lacZ::T7 gene1 [lon] ompT gal sulA11 R(mcr-73::miniTn10-TetS)2 [dcm] R(zgb-210::Tn10-TetS) endA1 Δ(mcrC-mrr)114::IS10*	New England Biolabs (C2566I, Ipswich, MA, USA)
DSM1313		*C. thermocellum*	Wild-type	DSMZ
AVM002	DSM1313	*C. thermocellum*	DSM1313 P_*clo1313_2638*_::ldh*	This study
AVM062	AVM002	*C. thermocellum*	DSM1313 P_*clo1313_2638*_::ldh* ∆*adhE* (*clo1313_1798*)	This study

*Contained a single T950A point mutation in the *ldh* gene resulting in an I317K amino acid substitution

### Plasmid construction

All plasmids used in this study are listed in Table 3. Deletion and integration plasmids were constructed as described previously [44]. DNA fragments were PCR amplified using pDGO145 or genomic DNA of *C. thermocellum* DSM1313 as template. Plasmids, PCR products, and genomic DNA were purified using commercially available kits from GeneJet (Thermo Fisher Scientific). Correct plasmid assembly was confirmed via diagnostic PCR and Sanger sequencing (Eurofins Genomics Sweden AB, Solna, Sweden) of the open reading frames, homologous flanks, and promoter regions. PCR for plasmid assembly and diagnostic PCR were performed using Phusion high-fidelity DNA polymerase and DreamTaq DNA polymerase (Thermo Fisher Scientific, Waltham, MA, USA), respectively, according to the manufacturer´s instructions with primers ordered from Thermo Fisher Scientific or Integrated DNA Technologies (IDT; Skokie, IL, USA) (Additional file 1).

**Table 3:** Plasmids used in this study

Plasmid name	Relevant characteristics	Accession number	Source
pDGO145	Deletion/Integration vector backbone	KY852359	[55]
pTK15*	*ldh* integration vector; Integrates *C. thermocellum ldh* downstream of the *clo1313_2638* promoter region	ON809520	This study
pTK25	*adhE* (*clo1313_1798*) markerless deletion vector	ON809521	This study

*Contained a single T950A point mutation in the *ldh* gene resulting in an I317K amino acid substitution

### Strain construction

*Clostridium thermocellum* transformations, selection, and counter selection were performed as described previously [54]. The native *C. thermocellum ldh* gene was integrated behind the *clo1313_2638* promoter in the wild-type strain DSM1313 using plasmid pTK15, resulting in strain AVM002. Deletion of *adhE* (*clo1313_1798*) in strain AVM002 using plasmid pTK25 yielded strain AVM062. Diagnostic PCR with DreamTaq DNA polymerase (Thermo Fisher Scientific) as well as Sanger sequencing (Eurofins Genomics Sweden AB) of the modified loci was used for genetic analysis. Diagnostic primers were designed to bind outside or inside the modified loci (Additional file 1). A single T950A point mutation in the *ldh* gene was detected in AVM002 and the daughter strain AVM062 and resulted in an I317K amino acid substitution for the last amino acid at the C-terminus of Ldh. This mutation was already present in plasmid pTK15, used to construct AVM002, and does not lie in or is close to a predicted substrate binding site, product release site, or allosteric site of Ldh [56]. Culture purity of constructed strains was routinely checked through Sanger sequencing (Eurofins Genomics Sweden AB) with 16S rRNA primers from IDT (Additional file 1).

### Cultivation and media

Physiological characterization of *C. thermocellum* was performed in batch cultures in 125-mL Wheaton serum bottles (DWK Life Sciences, Millville, NJ, USA) containing 50 mL modified low-carbon (LC) medium [45, 57] with 10 g L^−1^ cellobiose as carbon source and 2 g L^−1^ urea as nitrogen source. For cultivations with DSM1313 (wild-type) or AVM062 (P_*clo1313_2638*_::ldh* ∆*adhE*), 5 g L^−1^ or 10 g L^−1^ MOPS was used, respectively, to avoid excessive acidification of the fermentation broth. Serum bottles and stock solutions were prepared as described previously [44] with one modification. For experiments with added ethanol, ethanol was added to solution B containing Na_2_SO_4_, KH_2_PO_4_, and K_2_HPO_4_, at a concentration that was 1.25-fold higher than the final concentration. All other solutions (i.e., cellobiose, MOPS, urea, salts, vitamins, and trace elements) were added to solution B after sterilization to reach the final concentrations. Cultures were grown at 45, 50 or 55 °C and shaken at 180 rpm in a Jeio Tech ISS-4075R incubator shaker (Milmedtek AB, Karlskrona, Sweden).

Serum bottle cultures were inoculated from frozen glycerol stocks (− 80 °C), grown overnight, and transferred at an optical density at 600 nm [OD_600_] of 0.4 to 2.0 to fresh pre-heated precultures. After reaching the mid-exponential growth phase (OD_600_ between 0.7 and 1.5), samples from these cultures were used to inoculate pre-heated serum bottle cultures to an initial OD_600_ of 0.05. These serum bottles were used for growth studies in the presence of added ethanol and were sampled for OD_600_ and extracellular metabolite analysis (as described below) throughout the cultivation. Serum bottles used for precultures contained 5 g L^−1^ cellobiose and 0.5 g L^−1^ urea and were grown at 55 °C, while serum bottles used for growth studies in the presence of added ethanol contained 10 g L^−1^ cellobiose and 2 g L^−1^ urea and were grown at 45, 50, or 55 °C.

### Growth-arrest studies

Growth-arrest studies were performed using the sulfur-limited LC medium described previously [44]. To generate inocula for growth-arrest studies, frozen glycerol stocks were used to inoculate initial preculture serum bottles grown on modified LC medium [45, 57] with 5 g L^−1^ cellobiose and 0.5 g L^−1^ urea as described above. These cultures were used as inocula for fresh pre-heated precultures grown on 100 mL modified LC medium [45, 57] in 200-mL Kimble serum bottles (DWK Life Sciences) with 10 g L^−1^ cellobiose and 2 g L^−1^ urea. Cells were harvested from these exponentially growing cultures at an OD_600_ of 2.5 to 3.0. Harvesting and washing of the cells was done under anaerobic conditions using modified LC medium without cellobiose, cysteine, and Na_2_SO_4_ as described before [44]. After washing, cells were used to inoculate 125-mL Wheaton serum bottles (DWK Life Sciences), at an initial OD_600_ of 1.5 to 2.0, containing one of two modified LC media: LC medium (as control) and LC medium without Na_2_SO_4_ and with tenfold lower cysteine levels (0.01 g L^−1^). Both media contained 10 g L^−1^ cellobiose and 2 g L^−1^ urea. After inoculation, bottles were sampled regularly over the course of 48 to 72 h for both OD_600_ and extracellular metabolite analysis as described below.

### Analytic methods

Optical density and HPLC analysis of extracellular metabolites were performed as described before [44]. For calculation of the biomass yield on cellobiose (in g_x_ g^−1^) and the biomass-specific cellobiose fermentation rate (q_s,fermented_ in mmol g_x_^−1^ h^−1^), the cell dry weight (g_x_) was estimated from OD_600_ measurements using a previously determined conversion factor of 2.6 [44].

### Cell extract and lactate dehydrogenase assay

For in vitro enzyme activity assays of lactate dehydrogenase, cells were harvested from exponentially growing batch serum bottle cultures grown on LC medium with 5 g L^−1^ cellobiose. Cell-free extracts were prepared as described previously [45]. Lactate dehydrogenase was assayed at 55 °C in a Cary 50 UV–visible spectrophotometer equipped with a single-cell Peltier element (Varian AB, Solna, Sweden) by monitoring NADH oxidation at 340 nm over time [42]. The assay mixture (1 mL) contained 200 mM Tris–HCl (pH 7.3), 0.22 mM NADH, 1 mM fructose 1,6-bisphosphate, 10 mM pyruvate, and 50 or 100 µL cell-free extract. The reaction was started by the addition of pyruvate. Protein concentrations were quantified using a Bradford assay with bovine serum albumin as protein standard [58].

### Calculation of yields and biomass-specific rates

Yields on cellobiose (in g_x_ g^−1^ or mol mol^−1^) and maximum specific growth rates (µ^max^ in h^−1^) during serum bottle batch cultivations were calculated by plotting the product concentrations against the cellobiose concentration or the natural logarithm of OD_600_ against time, respectively, and using the absolute slopes of the resulting linear fit made by linear regression. Exponential growth was defined as the period for which the R^2^ of the linear fit of ln(OD_600_) against time was above 0.99 for a period covering at least two generations with at least four sample points, unless otherwise indicated.

The biomass-specific cellobiose fermentation rate (${q}_{s,fermented}$ in mmol g_x_^−1^ h^−1^) during growth-arrest studies was calculated from the biomass-specific cellobiose consumption rate and the biomass-specific glucose production rate (Eq. 1). It was assumed that all cellobiose which is not hydrolyzed to glucose can be used for formation of fermentation products. The specific cellobiose consumption rate and glucose production rate are calculated from the mass balance equations for cellobiose (Eq. 2) and glucose (Eq. 3). In these equations, ${C}_{s}$ denotes the residual cellobiose concentration (in mmol L^−1^), ${C}_{glu}$ is the glucose concentration (in mmol L^−1^), and ${C}_{x}$ is the biomass concentration (in g L^−1^).

1 $${q}_{s,fermented}\left(t\right)={q}_{s}\left(t\right)-0.5\cdot {q}_{glu}\left(t\right)$$

2 $${q}_{s}\left(t\right)=-\frac{d{C}_{s}\left(t\right)}{dt}\cdot \frac{1}{{C}_{x}\left(t\right)}$$

3 $${q}_{glu}\left(t\right)=\frac{d{C}_{glu}\left(t\right)}{dt}\cdot \frac{1}{{C}_{x}\left(t\right)}$$

When Eqs. 2 and 3 are substituted in Eq. 1, an expression for ${q}_{s,fermented}$ can be obtained (Eq. 4):

4 $${q}_{s,fermented}\left(t\right)=-\frac{1}{{C}_{x}\left(t\right)}\cdot \left(\frac{d{C}_{s}\left(t\right)}{dt}+0.5\cdot \frac{d{C}_{glu}\left(t\right)}{dt}\right)$$

Simplifying this equation yields an expression for ${q}_{s,fermented}$ as a function of the biomass concentration and the fermented cellobiose concentration (${C}_{s,fermented}\left(t\right)$) over time (Eq. 5):

5 $${q}_{s,fermented}\left(t\right)=-\frac{1}{{C}_{x}\left(t\right)}\cdot \frac{d{C}_{s,fermented}\left(t\right)}{dt}$$

The fermented cellobiose concentrations (${C}_{s,fermented}\left(t\right)$) were plotted against time in Microsoft Excel and fitted with a non-linear exponential decay model (Eq. 6) to calculate the derivative, $d{C}_{s,fermented}\left(t\right)/dt$.

6 $${C}_{s,fermented}\left(t\right)=\left({C}_{s,fermented,0}-{C}_{s,fermented,\infty }\right)\cdot {e}^{-kt}+{C}_{s,fermented,\infty }$$

where ${C}_{s,fermented,0}$ is the initial fermented cellobiose concentration (mmol L^−1^), ${C}_{s,fermented,\infty }$ is the fermented cellobiose concentration at infinite time (mmol L^−1^), and *k* is the rate constant (h^−1^). The Microsoft Excel Solver add-in was used to fit these parameters to the exponential decay model while minimizing the sum of squared errors between measured and model data. Division of the first derivative of Eq. 6 by the measured biomass concentration (${C}_{x}$) results in the specific cellobiose fermentation rate (Eq. 5; Additional file 5).

The biomass-specific cellobiose, glucose, acetate, and formate consumption or production rates were calculated in similar fashion by using mass balance equations and fitting the concentration data to a non-linear exponential decay model to determine $d{C}_{i}\left(t\right)/dt$. For the specific lactate production rate, the non-linear Gompertz equation was used (Eq. 7) to calculate $d{C}_{lactate}\left(t\right)/dt$.

7 $${C}_{lactate}\left(t\right)={C}_{lactate,\infty }\cdot {\left(\frac{{C}_{lactate,0}}{{C}_{lactate,\infty }}\right)}^{{e}^{-kt}}$$

where ${C}_{lactate,0}$ is the initial lactate concentration (mmol L^−1^), ${C}_{lactate,\infty }$ is the lactate concentration at infinite time (mmol L^−1^), and *k* is the rate constant (h^−1^) (Additional file 7).

### Data analysis

Unpaired Student´s *t-*test was used for comparison between values in this study.

## Supplementary Information

**Additional file 1: Table S1.** Primers used in this study.

**Additional file 2**: ** Figs. S1**–**S3.** Growth and product profiles of DSM1313 in the presence of 0–35 g L^−1^ added ethanol at 55, 50, and 45 °C.

**Additional file 3: Figs. S4**–**S6.** Growth and product profiles of AVM062 in the presence of 0–50 g L^−1^ added ethanol at 55, 50, and 45 °C.

**Additional file 4: Figs. S7–S9.** Growth and product profiles of DSM1313 during growth-arrest studies in the presence of 0–40 g L^−1^ added ethanol at 55, 50, and 45 °C.

**Additional file 5. **Calculations of biomass-specific fermentation rates of DSM1313 during growth-arrest studies.

**Additional file 6. Figs. S10**–**S14.** Biomass-specific product and consumption rates of DSM1313 as a function of time in the presence of 0–40 g L^−1^ added ethanol during growth-arrest studies.

**Additional file 7. **Calculations of biomass-specific consumption and production rates of DSM1313 during growth-arrest studies.

## Data Availability

All data generated or analyzed during this study are included in this published article [and its supplementary information files].

## References

[1] IRENA. Global Renewables Outlook: Energy transformation 2050: International Renewable Energy Agency; 2020. 292 p.

[2] IEA (2017). Technology roadmap—delivering sustainable bioenergy.

[3] Fulton LM, Lynd LR, Körner A, Greene N, Tonachel LR (2015). The need for biofuels as part of a low carbon energy future. Biofuels, Bioprod Biorefin.

[4] RFA. Annual Ethanol Production: Renewable Fuels Association; 2021. https://ethanolrfa.org/markets-and-statistics/annual-ethanol-production.

[5] Lynd LR, Beckham GT, Guss AM, Jayakody LN, Karp EM, Maranas C (2022). Toward low-cost biological and hybrid biological/catalytic conversion of cellulosic biomass to fuels. Energy Environ Sci.

[6] Lynd LR, Guss AM, Himmel ME, Beri D, Herring C, Holwerda EK (2016). Advances in consolidated bioprocessing using *Clostridium thermocellum* and *Thermoanaerobacter saccharolyticum*. Industrial biotechnology.

[7] Lynd LR, Liang X, Biddy MJ, Allee A, Cai H, Foust T (2017). Cellulosic ethanol: status and innovation. Curr Opin Biotechnol.

[8] Balch ML, Chamberlain MB, Worthen RS, Holwerda EK, Lynd LR (2020). Fermentation with continuous ball milling: effectiveness at enhancing solubilization for several cellulosic feedstocks and comparative tolerance of several microorganisms. Biomass Bioenerg.

[9] Balch ML, Holwerda EK, Davis MF, Sykes RW, Happs RM, Kumar R (2017). Lignocellulose fermentation and residual solids characterization for senescent switchgrass fermentation by *Clostridium thermocellum* in the presence and absence of continuous *in situ* ball-milling. Energy Environ Sci.

[10] Paye JMD, Guseva A, Hammer SK, Gjersing E, Davis MF, Davison BH (2016). Biological lignocellulose solubilization: comparative evaluation of biocatalysts and enhancement via cotreatment. Biotechnol Biofuels.

[11] Tindall BJ. The names *Hungateiclostridium* Zhang et al. 2018, *Hungateiclostridium thermocellum* (Viljoen et al. 1926) Zhang et al. 2018, *Hungateiclostridium cellulolyticum* (Patel et al. 1980) Zhang et al. 2018, *Hungateiclostridium aldrichii* (Yang et al. 1990) Zhang et al. 2018, *Hungateiclostridium alkalicellulosi* (Zhilina et al. 2006) Zhang et al. 2018, *Hungateiclostridium clariflavum* (Shiratori et al. 2009) Zhang et al. 2018, *Hungateiclostridium straminisolvens* (Kato et al. 2004) Zhang et al. 2018 and *Hungateiclostridium saccincola* (Koeck et al. 2016) Zhang et al. 2018 contravene Rule 51b of the International Code of Nomenclature of Prokaryotes and require replacement names in the genus *Acetivibrio* Patel et al. 1980. International Journal of Systematic and Evolutionary Microbiology. 2019;69:3927–32. 10.1099/ijsem.0.003685 31526446

[12] Holwerda EK, Worthen RS, Kothari N, Lasky RC, Davison BH, Fu C (2019). Multiple levers for overcoming the recalcitrance of lignocellulosic biomass. Biotechnol Biofuels.

[13] Tian L, Papanek B, Olson DG, Rydzak T, Holwerda EK, Zheng T (2016). Simultaneous achievement of high ethanol yield and titer in *Clostridium thermocellum*. Biotechnol Biofuels.

[14] Holwerda EK, Olson DG, Ruppertsberger NM, Stevenson DM, Murphy SJL, Maloney MI (2020). Metabolic and evolutionary responses of *Clostridium thermocellum* to genetic interventions aimed at improving ethanol production. Biotechnol Biofuels.

[15] Dien BS, Cotta MA, Jeffries TW (2003). Bacteria engineered for fuel ethanol production: current status. Appl Microbiol Biotechnol.

[16] Rogers PL, Jeon YJ, Lee KJ, Lawford HG. *Zymomonas mobilis* for Fuel Ethanol and Higher Value Products. Biofuels. 108. Berlin, Heidelberg: Springer Berlin Heidelberg; 2007. p. 263–88. 10.1007/10_2007_060 17522816

[17] Shi D-j, Wang C-l, Wang K-m (2009). Genome shuffling to improve thermotolerance, ethanol tolerance and ethanol productivity of *Saccharomyces cerevisiae*. J Ind Microbiol Biotechnol.

[18] Herrero AA, Gomez RF (1980). Development of ethanol tolerance in *Clostridium thermocellum*: effect of growth temperature. Appl Environ Microbiol.

[19] Tian L, Cervenka ND, Low AM, Olson DG, Lynd LR (2019). A mutation in the AdhE alcohol dehydrogenase of *Clostridium thermocellum* increases tolerance to several primary alcohols, including isobutanol, *n*-butanol and ethanol. Sci Rep.

[20] Williams TI, Combs JC, Lynn BC, Strobel HJ (2007). Proteomic profile changes in membranes of ethanol-tolerant *Clostridium thermocellum*. Appl Microbiol Biotechnol.

[21] Kundu S, Ghose TK, Mukhopadhyay SN (1983). Bioconversion of cellulose into ethanol by *Clostridium thermocellum*—product inhibition. Biotechnol Bioeng.

[22] Holwerda EK, Thorne PG, Olson DG, Amador-Noguez D, Engle NL, Tschaplinski TJ (2014). The exometabolome of *Clostridium thermocellum* reveals overflow metabolism at high cellulose loading. Biotechnol Biofuels.

[23] Thompson RA, Trinh CT (2017). Overflow metabolism and growth cessation in *Clostridium thermocellum* DSM1313 during high cellulose loading fermentations. Biotechnol Bioeng.

[24] Shao X, Raman B, Zhu M, Mielenz JR, Brown SD, Guss AM (2011). Mutant selection and phenotypic and genetic characterization of ethanol-tolerant strains of *Clostridium thermocellum*. Appl Microbiol Biotechnol.

[25] Brown SD, Guss AM, Karpinets TV, Parks JM, Smolin N, Yang S (2011). Mutant alcohol dehydrogenase leads to improved ethanol tolerance in *Clostridium thermocellum*. Proc Natl Acad Sci.

[26] Sudha Rani K, Seenayya G (1999). High ethanol tolerant *Clostridium thermocellum* Strains SS21 and SS22. World J Microbiol Biotechnol.

[27] Cray JA, Stevenson A, Ball P, Bankar SB, Eleutherio ECA, Ezeji TC (2015). Chaotropicity: a key factor in product tolerance of biofuel-producing microorganisms. Curr Opin Biotechnol.

[28] Casey GP, Ingledew WMM (1986). Ethanol tolerance in yeasts. CRC Crit Rev Microbiol.

[29] Ingram LO (1986). Microbial tolerance to alcohols: role of the cell membrane. Trends Biotechnol.

[30] Timmons MD, Knutson BL, Nokes SE, Strobel HJ, Lynn BC (2009). Analysis of composition and structure of *Clostridium thermocellum* membranes from wild-type and ethanol-adapted strains. Appl Microbiol Biotechnol.

[31] Burdette DS, Jung S-H, Shen G-J, Hollingsworth RI, Zeikus JG (2002). Physiological function of alcohol dehydrogenases and long-chain (C_30_) fatty acids in alcohol tolerance of *Thermoanaerobacter ethanolicus*. Appl Environ Microbiol.

[32] Tian L, Perot SJ, Stevenson D, Jacobson T, Lanahan AA, Amador-Noguez D (2017). Metabolome analysis reveals a role for glyceraldehyde 3-phosphate dehydrogenase in the inhibition of *C. thermocellum* by ethanol. Biotechnol Biofuels.

[33] Yang S, Giannone RJ, Dice L, Yang ZK, Engle NL, Tschaplinski TJ (2012). *Clostridium thermocellum* ATCC27405 transcriptomic, metabolomic and proteomic profiles after ethanol stress. BMC Genomics.

[34] Herrero AA, Gomez RF, Roberts MF (1985). 31P NMR studies of *Clostridium thermocellum*. Mechanism of end product inhibition by ethanol. J Biol Chem.

[35] Dash S, Olson DG, Joshua Chan SH, Amador-Noguez D, Lynd LR, Maranas CD (2019). Thermodynamic analysis of the pathway for ethanol production from cellobiose in *Clostridium thermocellum*. Metab Eng.

[36] Zheng T, Olson DG, Tian L, Bomble YJ, Himmel ME, Lo J (2015). Cofactor specificity of the bifunctional alcohol and aldehyde dehydrogenase (AdhE) in wild-type and mutant *Clostridium thermocellum* and *Thermoanaerobacterium saccharolyticum*. J Bacteriol.

[37] du Preez JC, Bosch M, Prior BA (1987). Temperature profiles of growth and ethanol tolerance of the xylose-fermenting yeasts *Candida shehatae* and *Pichia stipitis*. Appl Microbiol Biotechnol.

[38] Georgieva TI, Skiadas IV, Ahring BK (2007). Effect of temperature on ethanol tolerance of a thermophilic anaerobic ethanol producer *Thermoanaerobacter* A10: modeling and simulation. Biotechnol Bioeng.

[39] Sá-Correia I, Van Uden N (1983). Temperature profiles of ethanol tolerance: effects of ethanol on the minimum and the maximum temperatures for growth of the yeasts *Saccharomyces cerevisiae* and *Kluyveromyces fragilis*. Biotechnol Bioeng.

[40] Gao C, Fleet GH (1988). The effects of temperature and pH on the ethanol tolerance of the wine yeasts, *Saccharomyces cerevisiae*, *Candida stellata* and *Kloeckera apiculata*. J Appl Bacteriol.

[41] Redón M, Guillamón JM, Mas A, Rozès N (2011). Effect of growth temperature on yeast lipid composition and alcoholic fermentation at low temperature. Eur Food Res Technol.

[42] Lo J, Zheng T, Hon S, Olson DG, Lynd LR (2015). The Bifunctional Alcohol and Aldehyde Dehydrogenase Gene, *adhE*, Is Necessary for Ethanol Production in *Clostridium thermocellum* and *Thermoanaerobacterium saccharolyticum*. J Bacteriol.

[43] Olson DG, Maloney M, Lanahan AA, Hon S, Hauser LJ, Lynd LR (2015). Identifying promoters for gene expression in *Clostridium thermocellum*. Metab Eng Commun.

[44] Kuil T, Hon S, Yayo J, Foster C, Ravagnan G, Maranas CD (2022). Functional analysis of H^+^-pumping membrane-bound pyrophosphatase, ADP-glucose synthase, and pyruvate phosphate dikinase as pyrophosphate sources in *Clostridium thermocellum*. Appl Environ Microbiol.

[45] Yayo J, Kuil T, Olson DG, Lynd LR, Holwerda EK, van Maris AJA (2021). Laboratory evolution and reverse engineering of *Clostridium thermocellum* for growth on glucose and fructose. Appl Environ Microbiol.

[46] Ellis LD, Holwerda EK, Hogsett D, Rogers S, Shao X, Tschaplinski T (2012). Closing the carbon balance for fermentation by *Clostridium thermocellum* (ATCC 27405). Biores Technol.

[47] Biswas R, Zheng T, Olson DG, Lynd LR, Guss AM (2015). Elimination of hydrogenase active site assembly blocks H_2_ production and increases ethanol yield in *Clostridium thermocellum*. Biotechnol Biofuels.

[48] Riederer A, Takasuka TE, Makino S, Stevenson DM, Bukhman YV, Elsen NL (2011). Global gene expression patterns in *Clostridium thermocellum* as determined by microarray analysis of chemostat cultures on cellulose or cellobiose. Appl Environ Microbiol.

[49] Rydzak T, McQueen PD, Krokhin OV, Spicer V, Ezzati P, Dwivedi RC (2012). Proteomic analysis of *Clostridium thermocellum* core metabolism: relative protein expression profiles and growth phase-dependent changes in protein expression. BMC Microbiol.

[50] Lo J, Olson DG, Murphy SJ-L, Tian L, Hon S, Lanahan A (2017). Engineering electron metabolism to increase ethanol production in *Clostridium thermocellum*. Metab Eng.

[51] Jacobson TB, Korosh TK, Stevenson DM, Foster C, Maranas C, Olson DG (2020). In vivo thermodynamic analysis of glycolysis in *Clostridium thermocellum* and *Thermoanaerobacterium saccharolyticum* using ^13^C and ^2^H Tracers. mSystems..

[52] Olson DG, McBride JE, Joe Shaw A, Lynd LR (2012). Recent progress in consolidated bioprocessing. Curr Opin Biotechnol.

[53] Olson DG, Sparling R, Lynd LR (2015). Ethanol production by engineered thermophiles. Curr Opin Biotechnol.

[54] Olson DG, Lynd LR (2012). Transformation of *Clostridium thermocellum* by electroporation. Methods in enzymology.

[55] Hon S, Olson DG, Holwerda EK, Lanahan AA, Murphy SJL, Maloney MI (2017). The ethanol pathway from *Thermoanaerobacterium saccharolyticum* improves ethanol production in *Clostridium thermocellum*. Metab Eng.

[56] Wigley DB, Gamblin SJ, Turkenburg JP, Dodson EJ, Piontek K, Muirhead H (1992). Structure of a ternary complex of an allosteric lactate dehydrogenase from *Bacillus stearothermophilus* at 2·5 Å resolution. J Mol Biol.

[57] Holwerda EK, Hirst KD, Lynd LR (2012). A defined growth medium with very low background carbon for culturing *Clostridium thermocellum*. J Ind Microbiol Biotechnol.

[58] Bradford M (1976). A rapid and sensitive method for the quantitation of microgram quantities of protein utilizing the principle of protein-dye binding. Anal Biochem.

