# Supplementary material for: Ethanol tolerance of Clostridium thermocellum: the role of chaotropicity, temperature and pathway thermodynamics on growth and fermentative capacity
Source: Microb Cell Fact. 2022 Dec 25;21:273. doi: 10.1186/s12934-022-01999-8 (PMC9790125; doi:10.1186/s12934-022-01999-8)
Supplement: Supplementary file 1 — Additional file 1: Table S1. Primers used in this study. [file 12934_2022_1999_MOESM1_ESM.docx]

Table S1. Primers used in this study.

| **Number** | **Purpose** | **Sequence (5´ to 3´)*** |
| --- | --- | --- |
| 224 | Amplification of pDGO145 backbone | GATATCGCCTCGTGATACGC |
| 225 | Amplification of pDGO145 backbone | CAGCTGCTAATAGTAGTGAAAAAATCAG |
| 63 | Amplification of P*_gapDH_*-*cat*-*hpt* selection cassette from pDGO145 | GTGGGAATAGGCATGGAAAAGATTTTTTTGCC |
| 64 | Amplification of P*_gapDH_*-*cat*-*hpt* selection cassette from pDGO145 | GGGGAGGGCGTGAATGTAAGCGTGA |
| 379 | Amplification of 5´-flanking region (1) of *clo1313_2638* for pTK15 | ataaaaataggcgtatcacgaggcgatatcTCTGGATGCAATAAACAACGG |
| 237 | Amplification of 5´-flanking region (1) of *clo1313_2638* for pTK15 | gcaaaaaaatcttttccatgcctattcccacAACAAATTCCTCCTTACTTTTGTTATTATTC |
| 234 | Amplification of 5´-flanking region (2) of *clo1313_2638* for pTK15 | ttatgtcacgcttacattcacgccctccccTCTGGATGCAATAAACAACG |
| 89 | Amplification of 5´-flanking region (2) of *clo1313_2638* for pTK15 | gcaccaactacggttacttttttaattactttgttattgttcatAACAAATTCCTCCTTACTTTTG |
| 268 | Amplification of *ldh* for pTK15 | ATGAACAATAACAAAGTAATTAAAAAAGTAACCGTAGTTGGTGCAGGC |
| 269 | Amplification of *ldh* for pTK15 | TCATATATCTAGTGTTTTTATTATTTCTTTTAGAGTGTTTCCGGAC |
| 380 | Amplification of 3´-flanking region of P*_clo1313_2638_* for pTK15 | ctaaaagaaataataaaaacactagatatatgaATGTCACTGATTGGAACTGAAG |
| 231 | Amplification of 3´-flanking region of P*_clo1313_2638_* for pTK15 | ccctgattttttcactactattagcagctgGCCTTCTTTCCATTTTGCCG |
| 433 | Amplification of 5´-flanking region of *adhE* (*clo1313_1798*) for pTK25 | ataaaaataggcgtatcacgaggcgatatcCAAAGCTTGGATATGTTGCTG |
| 434 | Amplification of 5´-flanking region of *adhE* (*clo1313_1798*) for pTK25 | gttcttaccgctataaccttttcaagcttcatTTATTCGCTATTTTCGTCATTTTCC |
| 435 | Amplification of 3´-flanking region of *adhE* (*clo1313_1798*) for pTK25 | ggagataaggaaaatgacgaaaatagcgaataaATGAAGCTTGAAAAGGTTATAGCG |
| 436 | Amplification of 3´-flanking region of *adhE* (*clo1313_1798*) for pTK25 | gcaaaaaaatcttttccatgcctattcccacGTATATAAGGAGTGCCGTCTTTG |
| 437 | Amplification of internal region of *adhE* (*clo1313_1798*) for pTK25 | ttatgtcacgcttacattcacgccctccccGATACGGTAACACAGAAAGAG |
| 438 | Amplification of internal region of *adhE* (*clo1313_1798*) for pTK25 | ccctgattttttcactactattagcagctgGTGTCAGGCTGGAAAGCCAG |
| 282 | Confirmation of correct deletion plasmid assembly | GCCACCTGACGTCTAAGAAA |
| 281 | Confirmation of correct deletion plasmid assembly | AAGAAAACAGACGCGCCC |
| 280 | Confirmation of correct deletion plasmid assembly | GGAACCTTCCTTTTATAGGCG |
| 284 | Confirmation of correct deletion plasmid assembly | GTTAGAGCGGCATTATCCCT |
| 157 | Confirmation of correct deletion plasmid assembly | GGCAGCTAATAGAGGCATTA |
| 156 | Confirmation of correct deletion plasmid assembly | CCTAACTCTCCGTCGCTATT |
| 163 | Confirmation of correct deletion plasmid assembly | CCTGATGAATGAGTTGAGCTTC |
| 52 | Confirmation of correct plasmid assembly pTK15 | TGGGAATTCCCGCAATATTT |
| 59 | Confirmation of correct plasmid assembly pTK15 | ACGGCATGCCTTTTGTAAGGCCCGTT |
| 33 | Confirmation of P*_gapDH_*-*cat*-*hpt* selection cassette removal; Confirmation of correct plasmid assembly | GCTATCTTTACAGGTACATCATTCTGTTTGTG |
| 34 | Confirmation of P*_gapDH_*-*cat*-*hpt* selection cassette removal | TTTCATCAAAGTCCAATCCATAACCC |
| 285 | Confirmation of P*_cbp_*-*tdk* selection marker removal | ACGTTATATTGCTTGCCGGG |
| 289 | Confirmation of P*_cbp_*-*tdk* selection marker removal | AAGACTCCTTTGCTCCAACC |
| 273 | Confirmation of *ldh* integration downstream of the *clo1313_2638* promoter region | CCAACGGAATACCGGCAAAG |
| 274 | Confirmation of *ldh* integration downstream of the *clo1313_2638* promoter region | GGCGATCCGTCCCATACT |
| 53 | Confirmation of *ldh* integration downstream of the *clo1313_2638* promoter region; Confirmation of correct plasmid assembly pTK15 | GCCAACCAAAAAGAAGGCGA |
| 60 | Confirmation of *ldh* integration downstream of the *clo1313_2638* promoter region; Confirmation of correct plasmid assembly pTK15 | AACACTTTCATATATTTTATTCCGGGAAATCTGCTCC |
| 113 | Confirmation of *ldh* integration downstream of the *clo1313_2638* promoter region | GGTTCAGGGCTTCTGCTCAT |
| 115 | Confirmation of *ldh* integration downstream of the *clo1313_2638* promoter region | CCTACCTTTTTACAATGCAG |
| 272 | Confirmation of *ldh* integration downstream of the *clo1313_2638* promoter region | GCCGAATTTGCCGGAGAACG |
| 467 | Confirmation of *adhE* deletion | CAAAGCAGGACGGTGAATATG |
| 460 | Confirmation of *adhE* deletion; Confirmation of correct plasmid assembly pTK25 | CATAAGCTGCTCTAAGGTCTTTC |
| 459 | Confirmation of *adhE* deletion; Confirmation of correct plasmid assembly pTK25 | GTGCCGGCGGATGAAAATTC |
| 468 | Confirmation of *adhE* deletion | CAAATACTTGGCTGCCTTGTC |
| 1 | Amplification of 16S rRNA fragment for culture purity confirmation | AGAGTTTGATCCTGGCTCAG |
| 2 | Amplification of 16S rRNA fragment for culture purity confirmation | ACGGCTACCTTGTTACGACTT |
| *Uppercase letters indicate primer annealing sequences. Lowercase letters indicate the ≥ 30 bp overhang sequences used for Gibson assembly. | | |
