# Supplementary material for: Ethanol tolerance of Clostridium thermocellum: the role of chaotropicity, temperature and pathway thermodynamics on growth and fermentative capacity
Source: Microb Cell Fact. 2022 Dec 25;21:273. doi: 10.1186/s12934-022-01999-8 (PMC9790125; doi:10.1186/s12934-022-01999-8)
Supplement: Supplementary file 2 — Additional file 2: Figs. S1–S3. Growth and product profiles of DSM1313 in the presence of 0–35 g L−1 added ethanol at 55, 50, and 45 °C. [file 12934_2022_1999_MOESM2_ESM.docx]

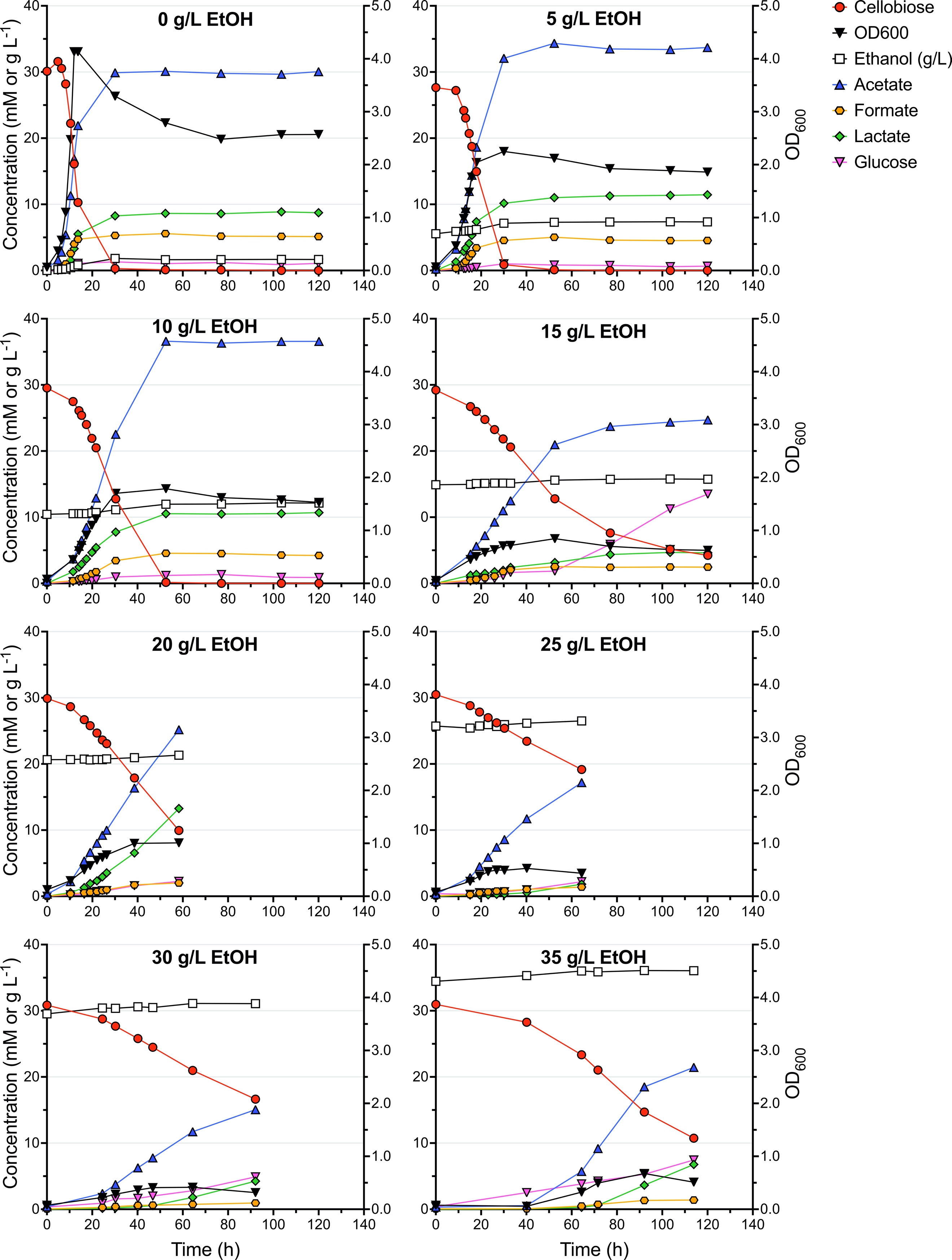


Fig. S1. Growth and product profiles of DSM1313 (wild-type) at 55 °C in the presence of various added ethanol concentrations. Batch serum bottle cultures were grown on modified LC medium with 10 g L^-1^ cellobiose. Data is shown for one representative experiment (*n* = 2).


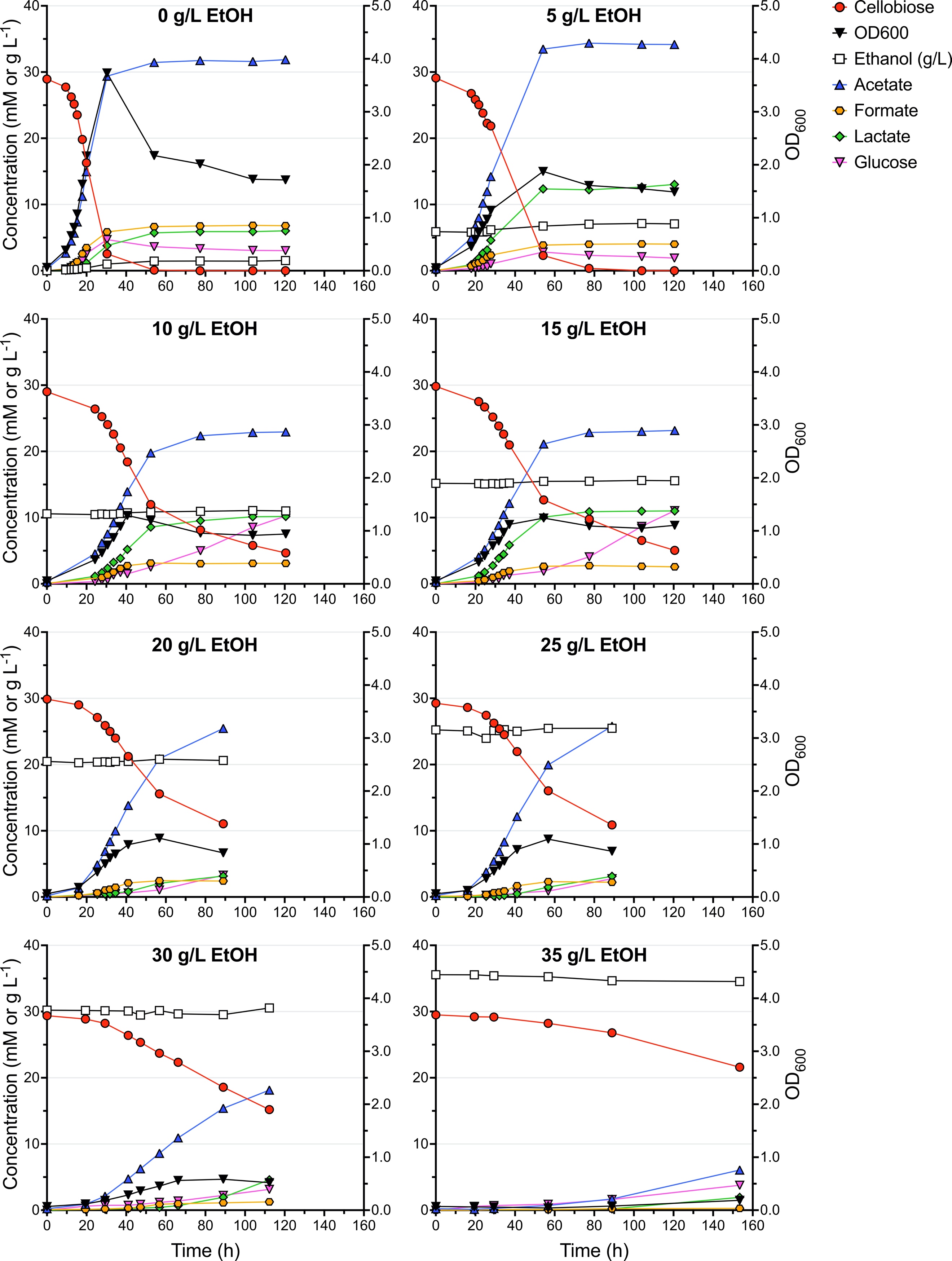


Fig. S2. Growth and product profiles of DSM1313 (wild-type) at 50 °C in the presence of various added ethanol concentrations. Batch serum bottle cultures were grown on modified LC medium with 10 g L^-1^ cellobiose. Data is shown for one representative experiment (*n* = 2).


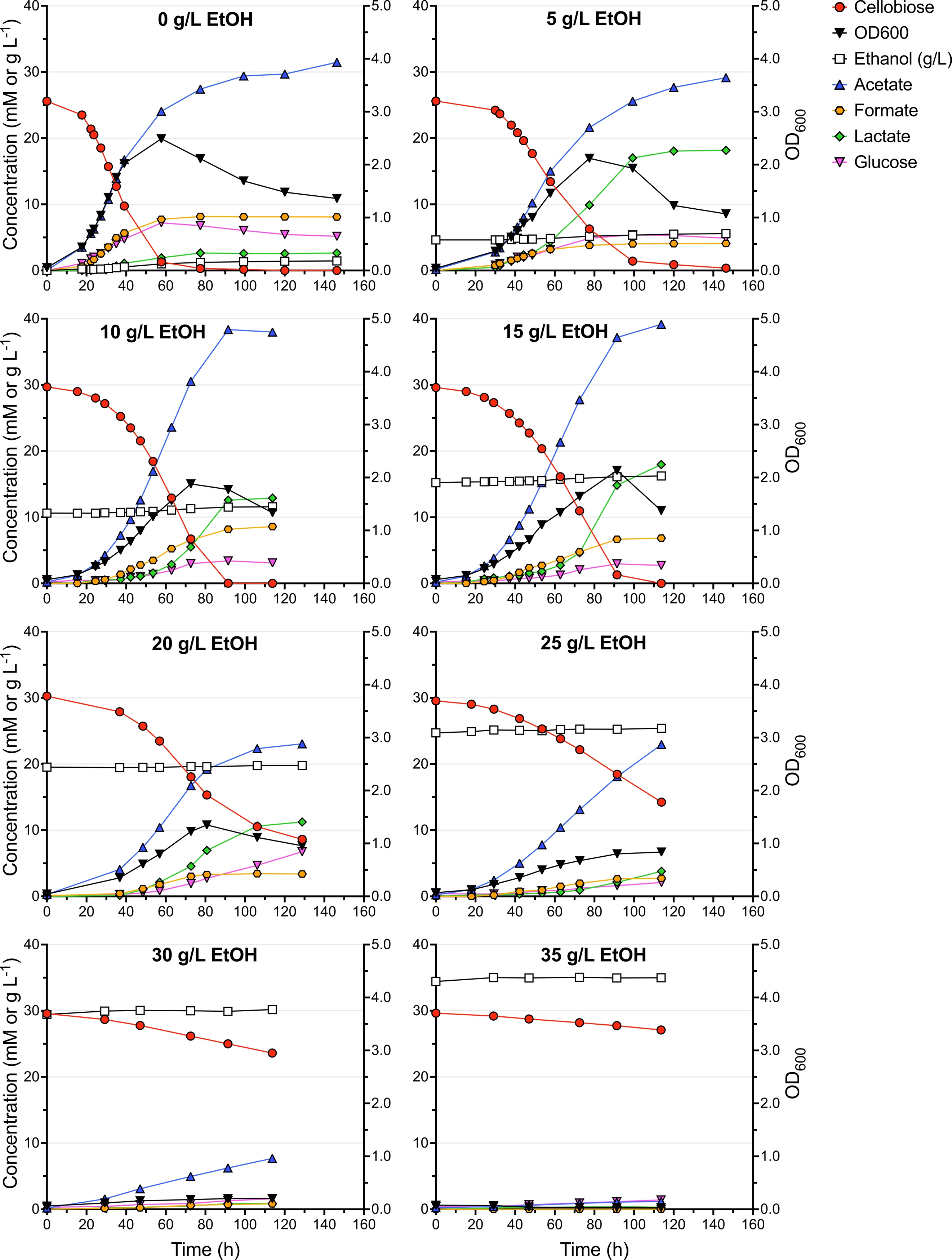


Fig. S3. Growth and product profiles of DSM1313 (wild-type) at 45 °C in the presence of various added ethanol concentrations. Batch serum bottle cultures were grown on modified LC medium with 10 g L^-1^ cellobiose. Data is shown for one representative experiment (*n* = 2).
