# Supplementary material for: Ethanol tolerance of Clostridium thermocellum: the role of chaotropicity, temperature and pathway thermodynamics on growth and fermentative capacity
Source: Microb Cell Fact. 2022 Dec 25;21:273. doi: 10.1186/s12934-022-01999-8 (PMC9790125; doi:10.1186/s12934-022-01999-8)
Supplement: Supplementary file 4 — Additional file 4: Figs. S7–S9. Growth and product profiles of DSM1313 during growth-arrest studies in the presence of 0–40 g L−1 added ethanol at 55, 50, and 45 °C. [file 12934_2022_1999_MOESM4_ESM.docx]

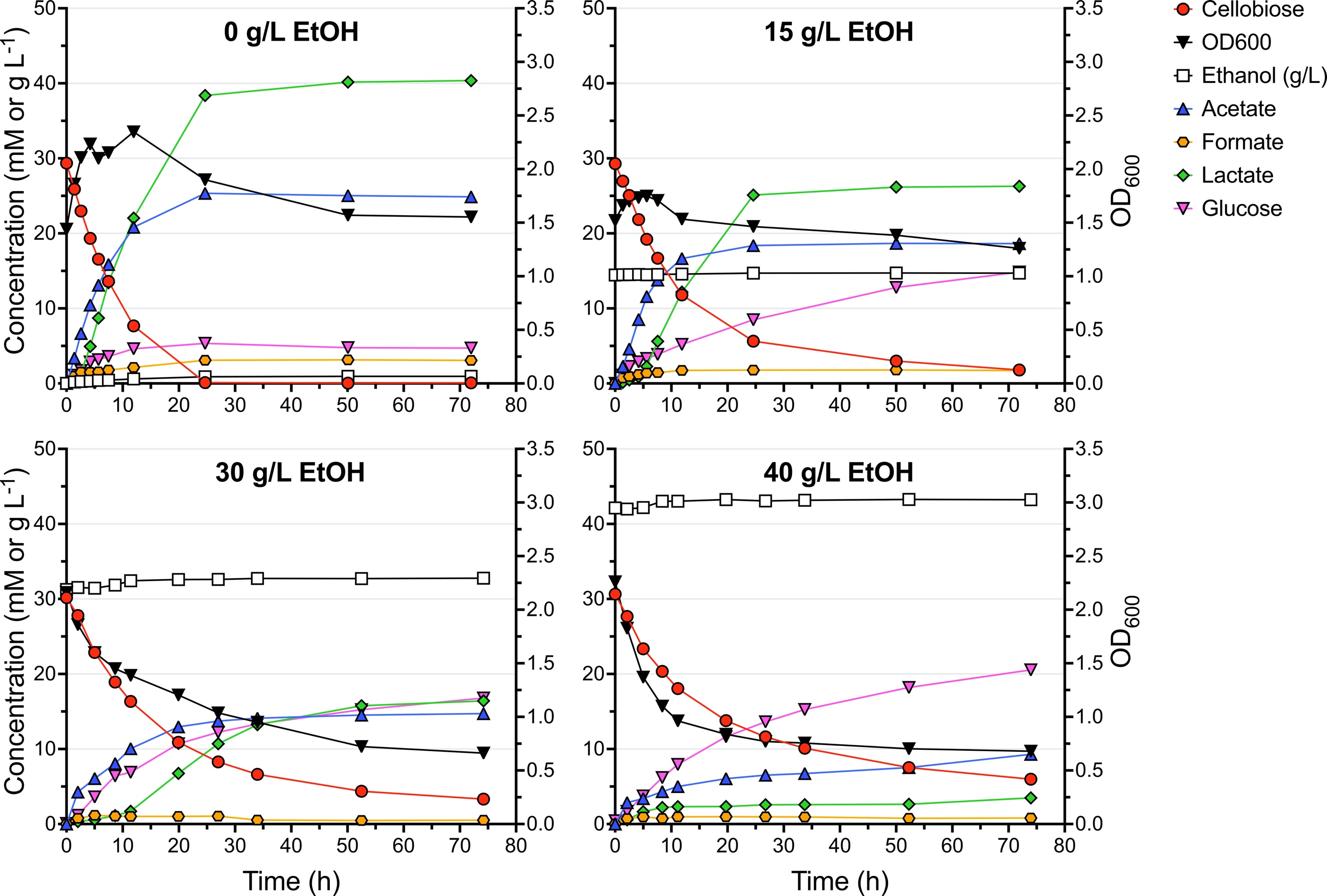


Fig. S7. Growth and product profiles of DSM1313 (wild-type) at 55 °C in the presence of various added ethanol concentrations during growth arrest studies. Batch serum bottle cultures were grown on modified LC medium without Na_2_SO_4_ and with 0.01 g L^-1^ cysteine and 10 g L^-1^ cellobiose. Data is shown for one representative experiment (*n* = 2).


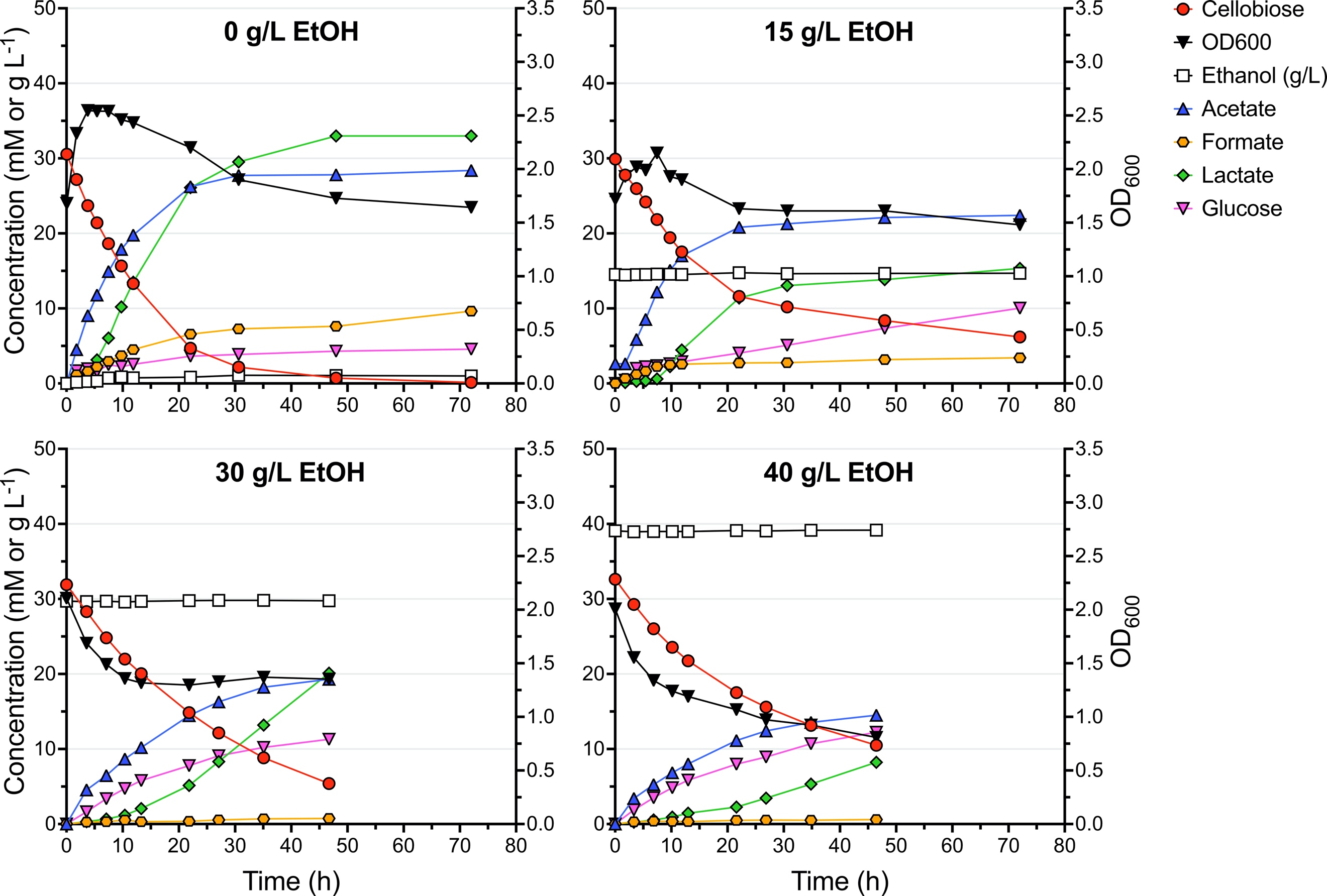


Fig. S8. Growth and product profiles of DSM1313 (wild-type) at 50 °C in the presence of various added ethanol concentrations during growth arrest studies. Batch serum bottle cultures were grown on modified LC medium without Na_2_SO_4_ and with 0.01 g L^-1^ cysteine and 10 g L^-1^ cellobiose. Data is shown for one representative experiment (*n* = 2).


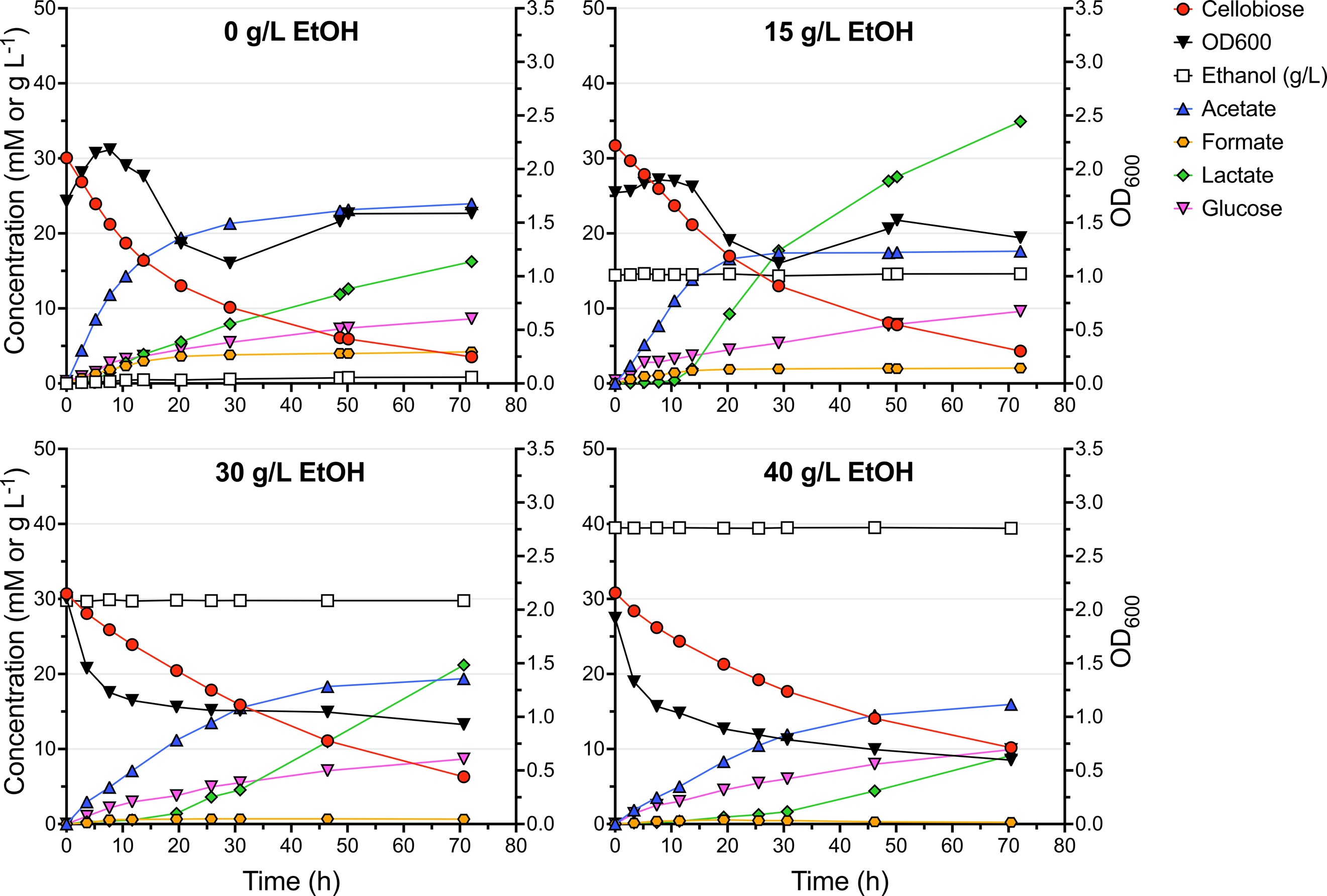


Fig. S9. Growth and product profiles of DSM1313 (wild-type) at 45 °C in the presence of various added ethanol concentrations during growth arrest studies. Batch serum bottle cultures were grown on modified LC medium without Na_2_SO_4_ and with 0.01 g L^-1^ cysteine and 10 g L^-1^ cellobiose. Data is shown for one representative experiment (*n* = 2).
