# Supplementary material for: Ethanol tolerance of Clostridium thermocellum: the role of chaotropicity, temperature and pathway thermodynamics on growth and fermentative capacity
Source: Microb Cell Fact. 2022 Dec 25;21:273. doi: 10.1186/s12934-022-01999-8 (PMC9790125; doi:10.1186/s12934-022-01999-8)
Supplement: Supplementary file 6 — Additional file 6. Figs. S10–S14. Biomass-specific product and consumption rates of DSM1313 as a function of time in the presence of 0–40 g L−1 added ethanol during growth-arrest studies. [file 12934_2022_1999_MOESM6_ESM.docx]

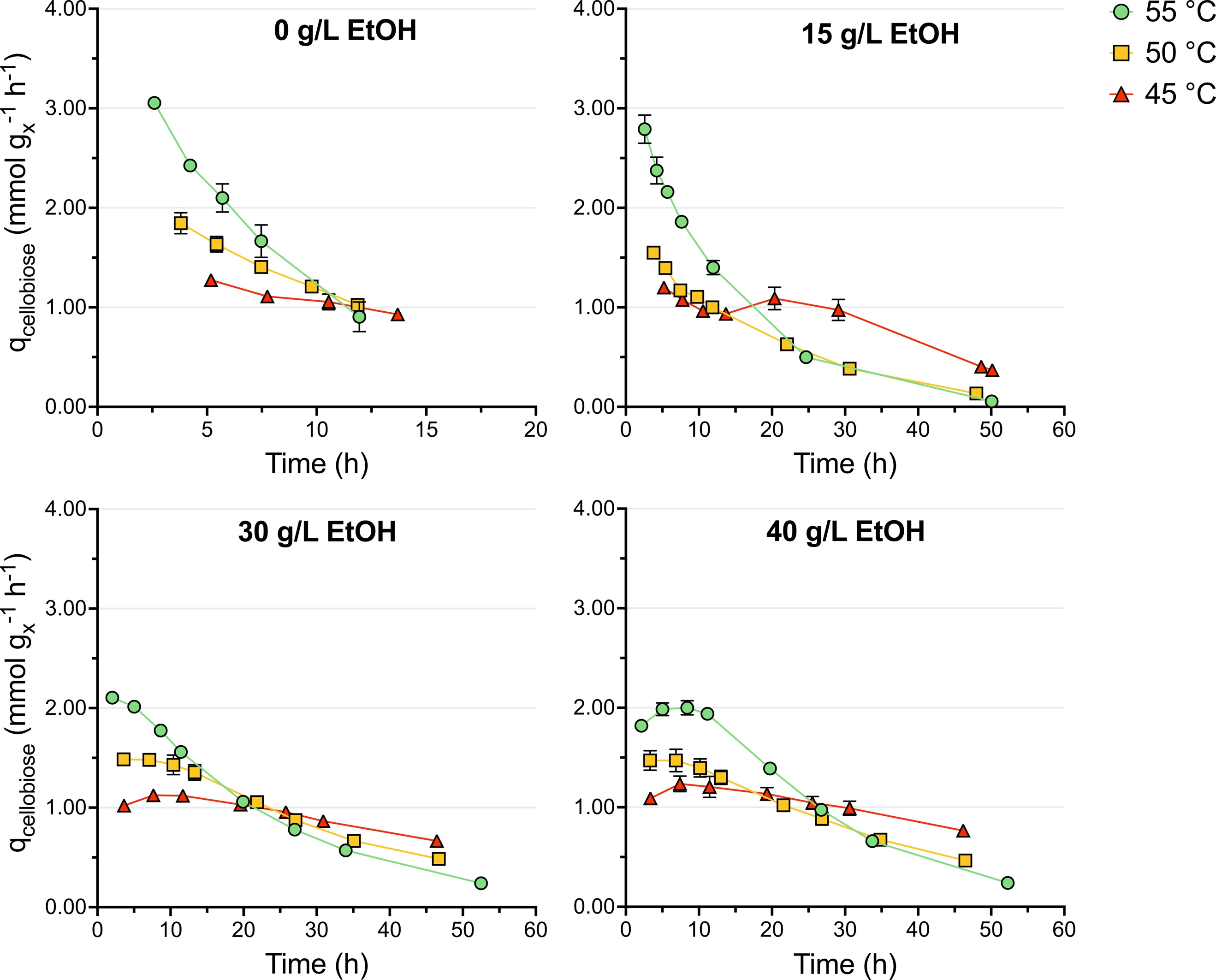


Fig. S10. Biomass-specific cellobiose consumption rate of DSM1313 as a function of time in the presence of 0, 15, 30, and 40 g L^-1^ added ethanol during growth-arrest studies. Batch serum bottle cultures were grown on modified LC medium without Na_2_SO_4_ and with 0.01 g L^-1^ cysteine and 10 g L^-1^ cellobiose. Data is plotted from the moment growth was arrested until cellobiose became limiting (> 0.5 mM) (Additional file 7). Averages and mean deviations were obtained from independent biological duplicates. Absence of error bars indicates mean deviations were smaller than the symbol size.


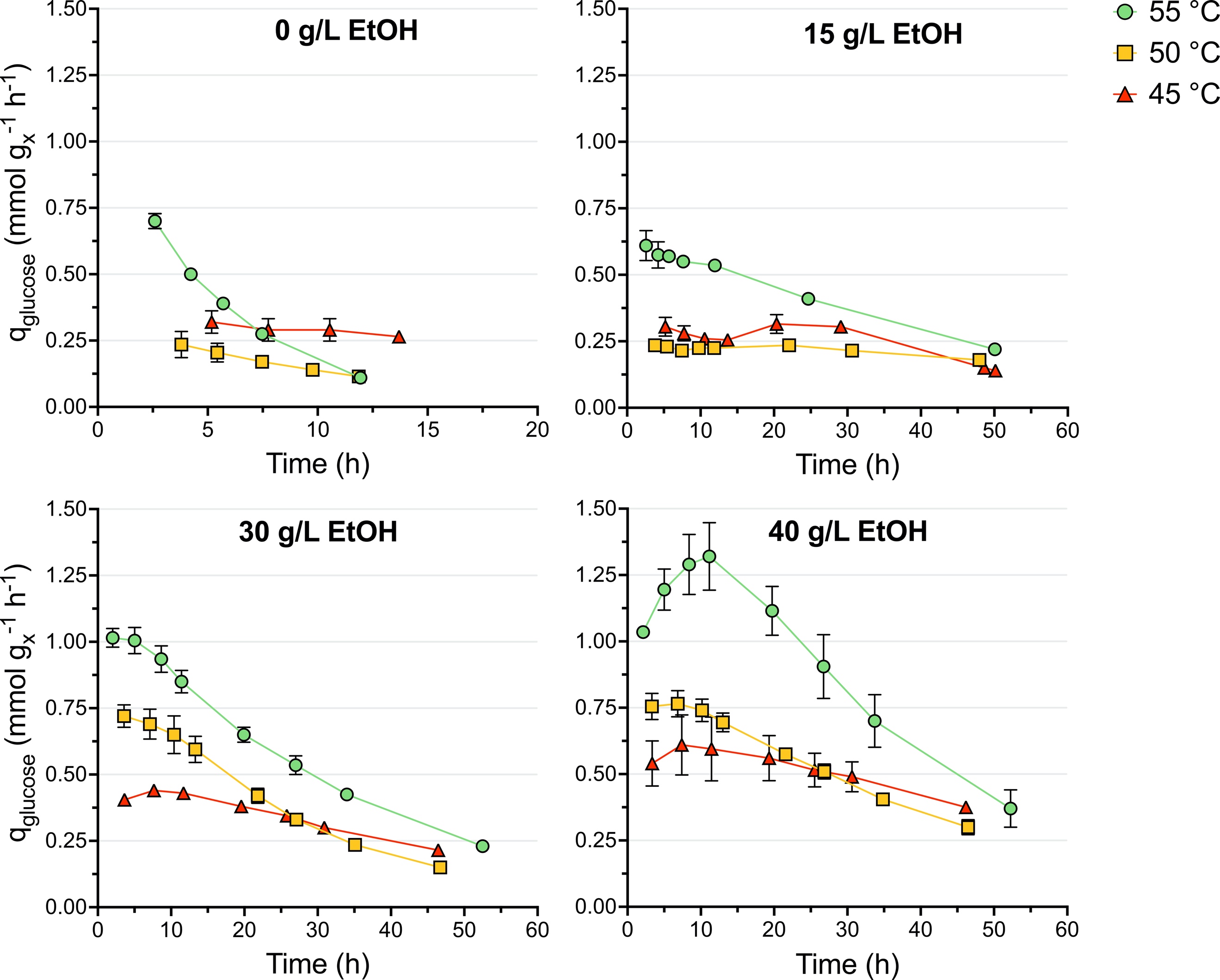


Fig. S11. Biomass-specific glucose production rate of DSM1313 as a function of time in the presence of 0, 15, 30, and 40 g L^-1^ added ethanol during growth-arrest studies. Batch serum bottle cultures were grown on modified LC medium without Na_2_SO_4_ and with 0.01 g L^-1^ cysteine and 10 g L^-1^ cellobiose. Data is plotted from the moment growth was arrested until cellobiose became limiting (> 0.5 mM) (Additional file 7). Averages and mean deviations were obtained from independent biological duplicates. Absence of error bars indicates mean deviations were smaller than the symbol size.


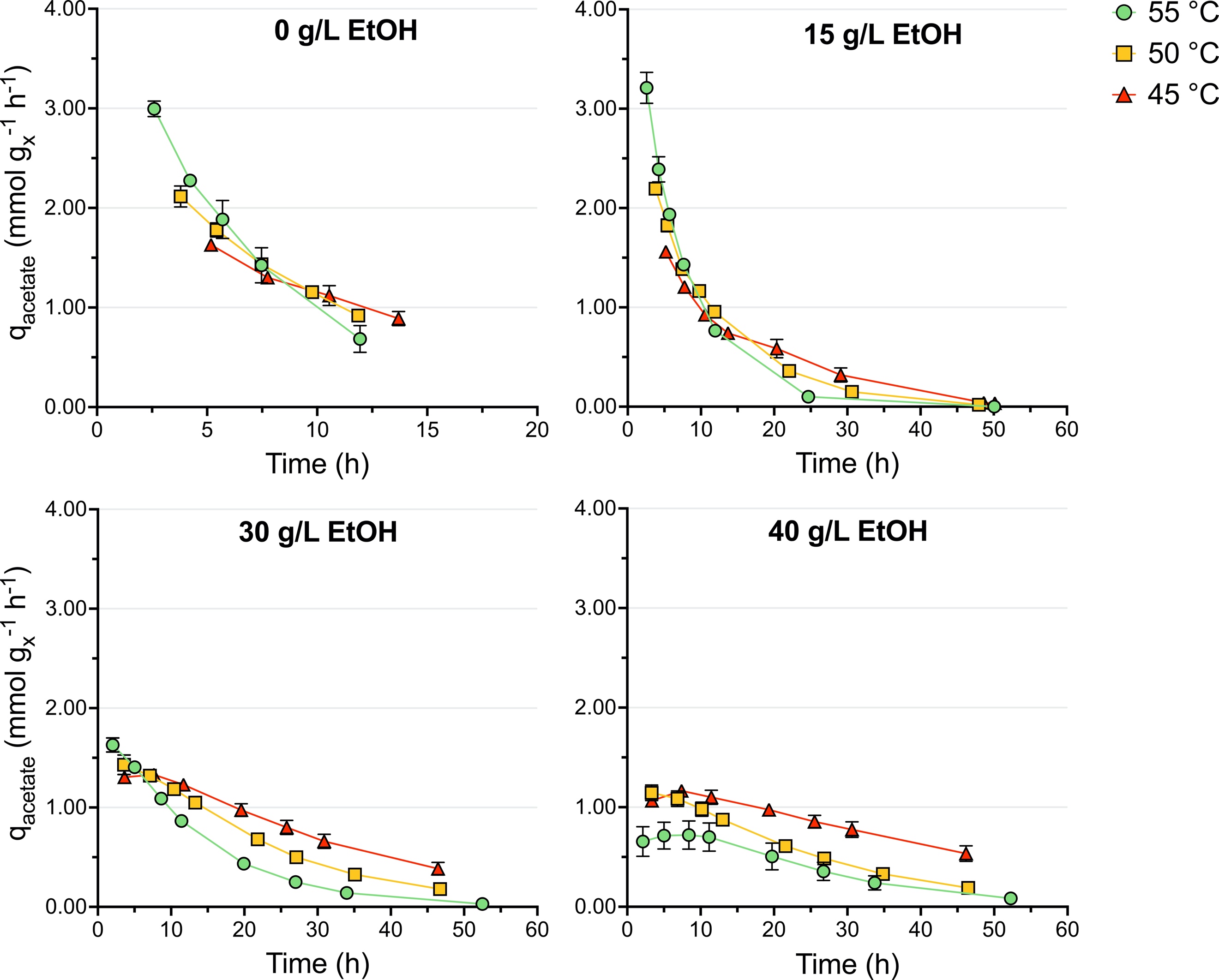


Fig. S12. Biomass-specific acetate production rate of DSM1313 as a function of time in the presence of 0, 15, 30, and 40 g L^-1^ added ethanol during growth-arrest studies. Batch serum bottle cultures were grown on modified LC medium without Na_2_SO_4_ and with 0.01 g L^-1^ cysteine and 10 g L^-1^ cellobiose. Data is plotted from the moment growth was arrested until cellobiose became limiting (> 0.5 mM) (Additional file 7). Averages and mean deviations were obtained from independent biological duplicates. Absence of error bars indicates mean deviations were smaller than the symbol size.


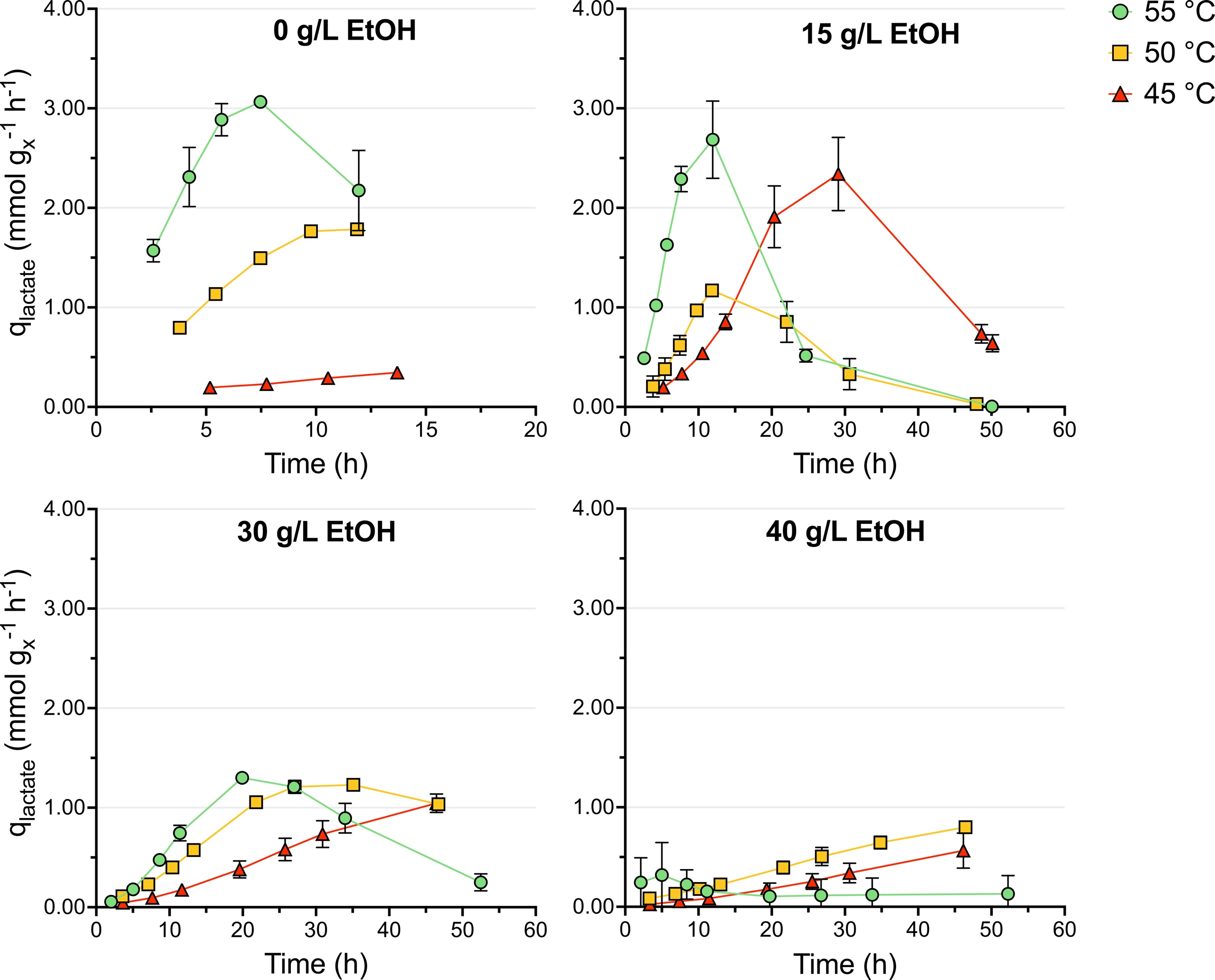


Fig. S13. Biomass-specific lactate production rate of DSM1313 as a function of time in the presence of 0, 15, 30, and 40 g L^-1^ added ethanol during growth-arrest studies. Batch serum bottle cultures were grown on modified LC medium without Na_2_SO_4_ and with 0.01 g L^-1^ cysteine and 10 g L^-1^ cellobiose. Data is plotted from the moment growth was arrested until cellobiose became limiting (> 0.5 mM) (Additional file 7). Averages and mean deviations were obtained from independent biological duplicates. Absence of error bars indicates mean deviations were smaller than the symbol size.


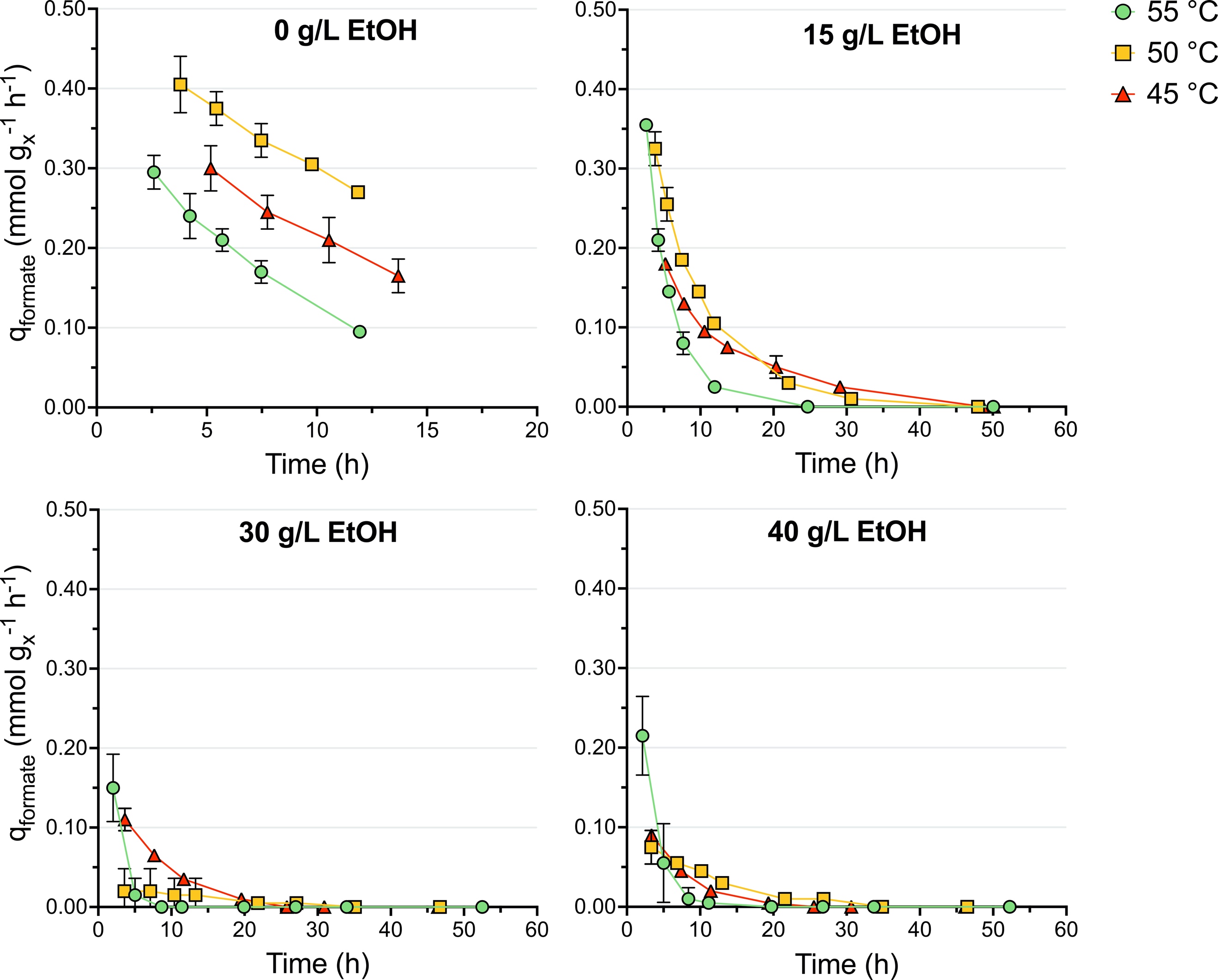


Fig. S14. Biomass-specific formate production rate of DSM1313 as a function of time in the presence of 0, 15, 30, and 40 g L^-1^ added ethanol during growth-arrest studies. Batch serum bottle cultures were grown on modified LC medium without Na_2_SO_4_ and with 0.01 g L^-1^ cysteine and 10 g L^-1^ cellobiose. Data is plotted from the moment growth was arrested until cellobiose became limiting (> 0.5 mM) (Additional file 7). Averages and mean deviations were obtained from independent biological duplicates. Absence of error bars indicates mean deviations were smaller than the symbol size.
